# Denaturing and dNTPs reagents improve SARS-CoV-2 detection *via* single and multiplex RT-qPCR

**DOI:** 10.12688/f1000research.109673.2

**Published:** 2024-03-28

**Authors:** Cristian E. Cadena-Caballero, Lina M. Vera-Cala, Carlos Barrios-Hernandez, Diego Rueda-Plata, Lizeth J. Forero-Buitrago, Carolina S. Torres-Jimenez, Erika Lizarazo-Gutierrez, Mayra Agudelo-Rodriguez, Francisco Martinez-Perez

**Affiliations:** 1 Grupo de Investigación Computo Avanzado y a Gran Escala - CAGE, Universidad Industrial de Santander, Bucaramanga, Santander, 680006, Colombia; 2 Grupo de Investigación en Demografía, Salud Pública y Sistemas de Salud - GUINDESS, Universidad Industrial de Santander, Bucaramanga, Santander, 680006, Colombia; 3 Centro de Supercomputación y Cálculo Científico de la Universidad Industrial de Santander -SC3UIS, Universidad Industrial de Santander, Bucaramanga, Santander, 680006, Colombia

**Keywords:** SARS-CoV-2, Diagnosis RT-qPCR, Adjuvant formulation, Primer and probe design, High performance computing

## Abstract

**Background:**

The COVID-19 pandemic, caused by the SARS-CoV-2, can be effectively managed with diagnostic tools such as RT-qPCR. However, it can produce false-negative results due to viral mutations and RNA secondary structures from the target gene sequence.

**Methods:**

With High Performance Computing, the complete SARS-CoV-2 genome was obtained from the GenBank/GISAID to generate consensus sequences to design primers/probes for RT-qPCR. *ORF8* gene was selected, meanwhile, *E* and *N* and *RNAse P* were according to CDC protocol. Nasopharyngeal swab samples were collected from patients diagnosed with SARS-CoV-2. Total RNA was purified according MagMAX kit, it was used in single, and multiplex RT-qPCR. To avoid templated secondary structures, compensate nucleotide proportions and improve Ct values, a solution composed of tetraethylammonium chloride and dimethyl sulfoxide and other with corresponding to dNTPs proportions in accordance SARS-CoV-2 genome were included. Sensitivity and specificity according to Ct values were determined with the Caret package in R software.

**Results:**

126,576 SARS-CoV-2 genomes from January to December 2020 comprised a database. From this, a region near of 5′ *ORF8* gene showed three stem-loops was used for primers/FAM-probe. 49 samples were obtained, from them, 22 were positive to gene selected regions. Interestingly, samples from October to November 2020 were positive for all regions, however, in January 2021 different results were observed in *ORF8*. An improvement in Ct with the adjuvant solutions was determined in all samples with others SARS-CoV-2 primers/probes, for both single and multiplex RT-qPCR. The inclusion of the denaturing solution in single RT-qPCR increased its sensitivity with respect to the commercial method, while in multiplex the opposite was generated.

**Conclusions:**

Including adjuvant solutions to prevent the formation of RNA secondary structures and the adjustment of the nucleotide ratios of SARS-CoV-2 improved single and multiplex RT-qPCR for viral identification, demonstrating its potential application in health public.

## Introduction

On January 23, 2020, the SARS-CoV-2 virus ( *Coronaviridae*: *Betacoronavirus*: *Severe acute respiratory syndrome-related coronavirus*) was declared a public health emergency by the World Health Organization (WHO) International Health Regulations (IHR) Emergency Committee. At the time, the global public health authorities established that the spread of SARS-CoV-2 could be prevented if every nation adopted solid strategies for rapid and accurate disease detection ( WHO, 2020).

Diagnostic methods based on gene-specific primers and probes for the detection of viruses *via* gene amplification include quantitative real-time polymerase chain reaction (RT-qPCR) or reverse transcription loop-mediated isothermal amplification (RT-LAMP), both of which can be conducted using oropharyngeal and nasopharyngeal swabs samples from patients ( Kevadiya *et al.*, 2021). Among these, the former method is considered the most sensitive and accurate for the detection of SARS-CoV-2 and other viruses ( Martín *et al.*, 2021). This procedure could be effectively implemented due to the characterization of the viral genome, which encompasses approximately 10 genes ( Zhu *et al.*, 2020). Therefore, WHO authorized the Berlin protocol which relies on the following genes: *ORF1ab*, which encodes proteins that enable viral replication ( Nur *et al.*, 2015); *Spike* ( *S*), which interacts with the receptor of the host’s angiotensin-converting enzyme 2 (ACE2) ( Wan *et al.*, 2020); and *E*, which encodes the structural envelop protein ( Tahan *et al.*, 2021). Other protocols such as the 2019-nCoV TaqMan RT-qPCR Kit authorized by the United States Centers for Disease Control and Prevention (CDC) utilize two regions of the *N* gene (N1-N2), which encode for the nucleocapsid phosphoprotein ( Navarathna *et al.*, 2021). Moreover, both protocols use the *RNase P* gene as a control to assess the efficiency of the RT-qPCR protocol ( WHO, 2009).

Nevertheless, RT-qPCR-based diagnosis is conducted on a per-gene basis and therefore its widespread implementation would be both impractical and costly. Therefore, multiplex RT-qPCR ( *e.g.*, the CDC kit or combined quantification of the *ORF1ab* and *S* genes) could be implemented as a promising approach to meet the current demand for accurate and cost-efficient diagnosis ( Kudo *et al.*, 2020). Sample pooling is another approach that could increase population-wide SARS-CoV-2 diagnosis rates and has therefore been approved and implemented in several countries ( Grobe *et al.*, 2021). However, sample numbers and sampling structure may limit the implementation of this strategy ( Grobe *et al.*, 2021).

Although RT-qPCR-based SARS-CoV-2 diagnostic tools are generally considered the gold standard for disease detection, several studies have determined that this approach is prone to return false-negative results due to gene mutations (which is often the case for the *E* and *N* genes) ( Hasan *et al.*, 2021; Tahan *et al.*, 2021) or primer dimer formation ( Jaeger *et al.*, 2021). Another factor that affects RT-qPCR efficiency is the secondary structure of the RNA to be characterized/quantified ( Hammerling *et al.*, 2020).

Although previous studies have confirmed the influence of SARS-CoV-2 RNA secondary structure on evolutionary dynamics and transcript regulation, among other factors ( Andrews *et al.*, 2021; Huston *et al.*, 2021; Rangan *et al.*, 2020; Wacker *et al.*, 2020), previous studies have not established whether this phenomenon directly or indirectly affects RT-qPCR efficiency using patient-derived samples. Moreover, the effects of adjuvants such as DMSO or ammonium salts on RT-qPCR efficiency and viral detection are also largely unknown ( Kovarova & Draber, 2000). Therefore, using high-performance computing (HPC), we consolidated a global repository of SARS-CoV-2 genomes published until December 2020, which we then used to select a region of the *ORF8* gene for both single and multiplex RT-qPCR-based viral detection using the *E* gene (Berlin protocol) and the *N* gene (CDC protocol) as targets. Furthermore, our findings demonstrated that adjusting the TMCA-DMSO and dNTP proportions of the denaturing solutions based on the nucleotide composition of the virus eliminates the secondary RNA structures that adversely affect the reaction. The detection and diagnosis implications of our findings will be discussed below.

## Methods

### Ethics statement

This study was approved by the Research Ethics Committees of the Industrial University of Santander, the Chicamocha Clinic, and the Chicamocha Clinical Laboratory (L3C) (Bucaramanga, Santander, Colombia). The study participants were patients either hospitalized-diagnosed with SARS-CoV-2 or persons that presented to the emergency room or volunteers. All participants provided written informed consent and voluntarily participated in our study. Further, all participants were kept anonymous and were informed that the present study was conducted strictly for research purposes and that its outcomes were not intended to serve as treatment or diagnosis.

### SARS-CoV-2 sample collection and storage

Nasopharyngeal swab samples were acquired by L3C personnel. Two samples were obtained per study participant, one for clinical diagnosis, as authorized by the Ministry of Health and Social Protection of Colombia, and the other for our study. The samples were placed in Universal Transport Medium (UTM) and stored at −80°C until required for downstream analyses ( Rogers *et al.*, 2020). The samples were collected over the course of one week and then appropriately packaged according to the sanitary legislation of Colombia ( Ministry of Health and Social Protection, 2020) prior to their transportation and processing. All samples were shipped to the Central Research Laboratory of the Industrial University of Santander Health Faculty (LCI-FS-UIS) and maintained at 4°C while the informed consents were processed and digitalized.

Afterwards, the samples were aliquoted in a Class II, Type A2 Biosafety Cabinet (Thermo Fisher Scientific). A total of three aliquots were obtained from each sample, including a 200-μL aliquot for total RNA extraction, which was used immediately, and two 650-μL aliquots used as backup samples, which were stored at −80°C.

### SARS-CoV-2 genome database consolidation

Due to the appearance of mutations in several genes of the SARS-CoV-2 reference genome that could generate false negatives with the primers and probes authorized for identification by RT-qPCR. A region coding for the accessory protein ORF8 was selected, hypothesizing that it did not have a high mutation frequency. Therefore, monthly consensus sequences were generated to determine its identity pattern with respect to new genomes. Publicly available SARS-CoV-2 genome sequences were obtained from the GenBank and GISAID databases (RRID:SCR_002760; RRID:SCR_018251) ( Sayers *et al.*, 2021; Shu & McCauley, 2017). To gather data from the GenBank database, a Python script (RRID:SCR_008394) was executed in the GUANE-1 High Performance and Scientific Computing Center of the Industrial University of Santander (SC3UIS) through the Entrez Programming Utilities interface using the Biopython package (RRID:SCR_013249; RRID:SCR_007173) ( Cock *et al.*, 2009; Geer *et al.*, 2010), whereas all GISAID data were manually downloaded. Genomes with uncharacterized regions/nucleotides were discarded using another Python script (RRID:SCR_008394), which was used to conduct monthly FASTA sequence alignments using the MAFFT software (RRID:SCR_011811) ( Katoh & Standley, 2013) coupled with previously described DNA loss model parameters ( Martínez-Pérez *et al.*, 2002, 2007). The consensus sequences were generated using BioEdit version 7.2 (RRID:SCR_007361) with a 100% threshold frequency ( Hall, 1999).

### Design and synthesis of *ORF8*-specific primers, probes, and substrate oligos

Using the SARS-CoV-2 (NC_045512) reference genome and consensus sequences from January to April 2020 ( Zhu *et al.*, 2020), an approximately 150-base pair (bp) region of the *ORF8* gene was selected based on the secondary structure of its RNA transcript, which in turn was predicted using the algorithms proposed by Zuker ( Zuker & Jacobson, 1998) via the Mflod software (RRID:SCR_001360) ( Zuker, 2003). Thus, the last set containing the codons of the central region of the *ORF8* gene, which encodes the secretion protein ORF8, was chosen because it allows proper viral adhesion to the host cell ( Chan *et al.*, 2020). The obtained sequence was used as a template to create primers, a TaqMan FAM-BBQ probe, and substrate oligos for RT-qPCR. The specificity of the aforementioned molecules was confirmed via GenBank BLAST analyses (RRID:SCR_004870) ( Ye *et al.*, 2006). Genes *E* and *N* from the Berlin and CDC protocols were used as controls ( Biotek, 2020; Corman *et al.*, 2020).

All molecules were synthesized by Bioneer (Korea). The primers were purified *via* separation on a reverse-phase cartridge, whereas the probe and substrate oligos were purified *via* high-performance liquid chromatography (HPLC) and polyacrylamide gel electrophoresis (PAGE), respectively. The *ORF8* RT-PCR conditions were implemented as described by the Berlin protocol ( Corman *et al.*, 2020) using the aforementioned molecules coupled with the 2019-nCoV TaqMan RT-PCR Kit (Norgen Biotek Corp) developed by the CDC ( Biotek, 2020).

### Preparation of denaturing solutions and adjustment of dNTP concentrations

A:T and G:C ratios were calculated based on the monthly SARS-CoV-2 consensus sequences to obtain an average for each nucleotide. These averages were then used to determine the minimum concentrations of TEA (ABCAM-USA), DMSO (Scharlab-Spain), and dNTPs (100 mM each, Promega-USA) in molecular-grade ultrapure water (Promega-USA), in addition to the MgSO _4_ concentration recommended by the Berlin protocol.

### RNA extraction

Total RNA extraction was conducted using the MagMAX™ Viral/Pathogen II (MVP II) Nucleic Acid Isolation Kit (2000 RXNs) (Applied Biosystems-USA) using a KingFisher Duo Prime (5400110) DNA/RNA extraction system according to the manufacturer’s instructions (Thermo Fisher Scientific-USA) ( Fang *et al.*, 2007).

### SARS-CoV-2 single and multiplex RT-qPCR

RT-qPCR was conducted using the *ORF8*-specific primers and probe designed herein, in addition to the *E* (Berlin protocol) and *N* genes (N1-N2; CDC protocol). The *RNase P* gene was used as an external control, as proposed by both of the aforementioned protocols. The reactions were conducted using the SuperScript III One-Step RT-PCR System with Platinum Taq DNA Polymerase (Ref. 11732088; Invitrogen-USA) and the 2019-nCoV TaqMan RT-PCR Kit (Ref. TM67100; Norgen-Biotek-Canada). Each reaction for each diagnostic system was conducted in either 15- or 25-μL reaction volumes consisting of 2 μL of patient-derived purified RNA, 2× One-Step RT-PCR Master Mix, 2× nuclease-free buffer, and the respective primers/probe at the concentrations recommended by the CDC. Moreover, a denaturing stock solution was added to obtain a final concentration of 0.7% TEA, 0.2% DMSO, and 0.8 mMol MgSO _4_. The dNTP reagent had a final concentration of 12 mMol dATP-dTTP, 10 mMol dCTP-dGTP, and 0.8 mMol MgSO _4_. The reaction conditions were the following: 55°C for 15 minutes, 95°C for 3 minutes followed by 45 cycles of 95°C for 15 s and 58°C for 30 s for the SuperScript™ III One-Step RT-PCR kit; and 95°C for 3 s followed by 55°C for 20 s for the 2019-nCoV TaqMan RT-PCR kit. Fluorescence signals were quantified using a QuantStudio 1 Real-Time PCR System (No. A40427) in a 96-well 0.2 μL block (Thermo Fisher Scientific-USA).

The above-described procedure was conducted using two different Multiplex One-Step RT-qPCR protocols. In the first instance, the primers and probes for the *E*, *ORF8*, and *N* (N1) genes were mixed, whereas the other reaction was performed by mixing the N1 and N2 sets of the *N* gene. The *RNase P* gene was independently assessed in both cases. All reactions were performed as described above.

### Calculation of sensitivity, specificity and predictive values of SARS-CoV-2

To calculate the sensitivity, specificity, Positive Predictive Value (PPV) and Negative Predictive Value (NPV) of each of the reactions with the single and multiplex primers used, a script was implemented in R using the Caret package to calculate the values generated by each RT-qPCR with their respective primers/probes ( https://github.com/GenomicUIS/Sensitivity-specificity-PPV-and-NPV-for-SARS-CoV-2.git) ( Kuhn, 2008; R Core Team 2020). Cycle threshold (Ct) was defined for each of the primers in the single reactions of each of the processed samples, where, the value of 18-35 Ct was considered true positive, Ct 35 > false positive, Ct 18 < true negative. Subsequently, the Cts values obtained were pooled and evaluated per sample. Therefore, if the result generated by a set of primers/probes was at the above-mentioned threshold, it was considered a true positive identification. Whereas, results outside the threshold were considered false negative and indeterminate. Cts were identical for multiplex RT-qPCR reactions, but the results were evaluated as a whole.

## Results

### SARS-CoV-2 GenBank and GISAID databases

A total of 19,317 genomes were retrieved from the GenBank database from January to October 2020 based on our search criteria, whereas the GISAID database rendered 107,259 full genomes from January to December 2020. In both cases, the number of available sequences increased substantially each month. However, the frequency of base substitutions in the monthly consensus sequences for the *E*, *ORF8*, and *N* genes was higher in the GISAID database compared to the GenBank database ( Figure 1).

**Figure 1.**
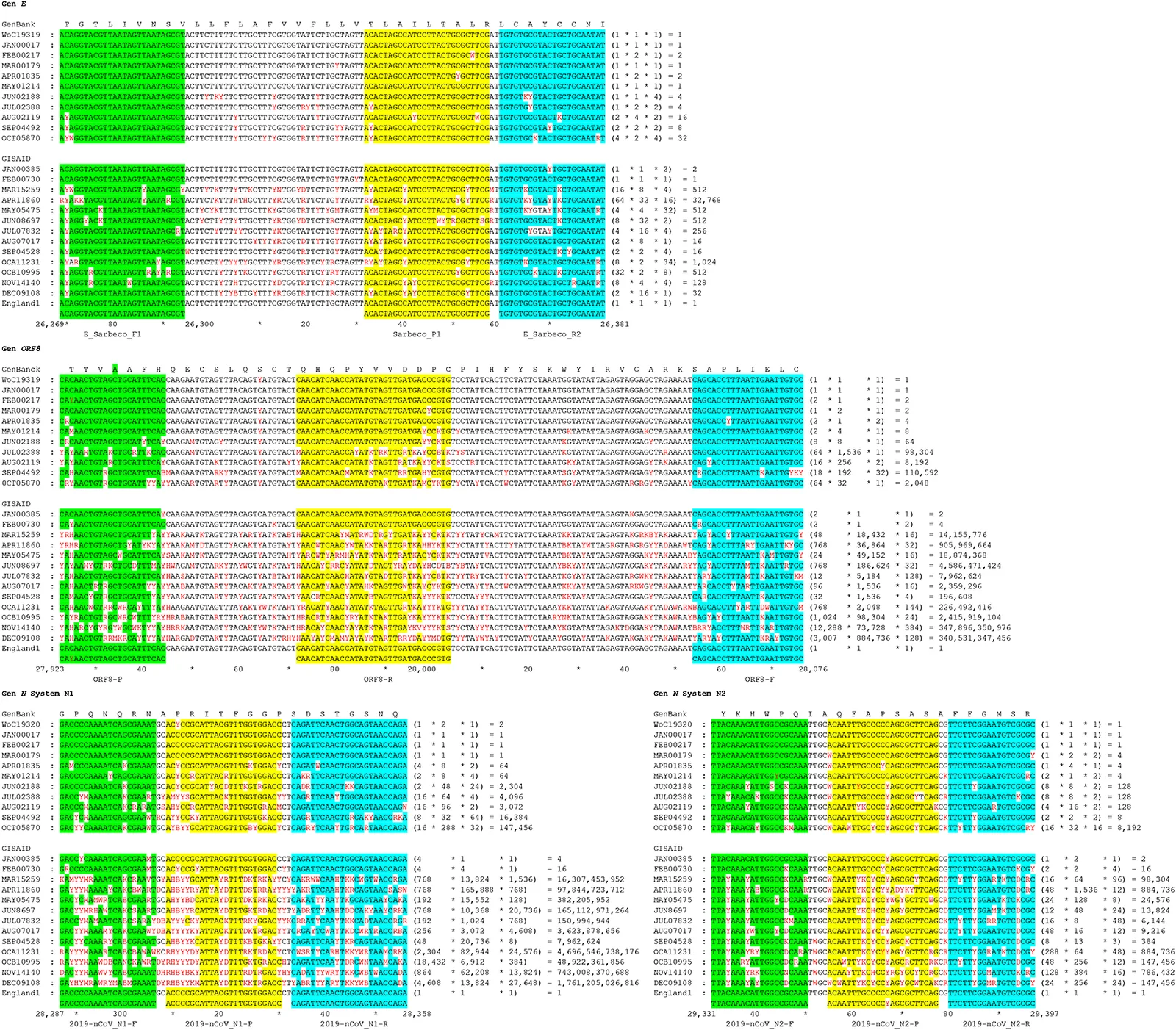
Primers and probes to RT-qPCR to SARS-CoV-2. The nucleotides correspond to: forward primer in green box and lower primer blue box. The Probes is in yellow box. The conceptual translation is indicated in the top of each alignment. The number indicate the nucleotide combination. The nucleotides values combination by position corresponds to international nomenclature. The nucleotide position according to SARS-CoV-2 reference genome is indicated in the lower consensus alignment.

### Primer, probe, and substrate oligo design for SARS-CoV-2 detection based on RNA secondary structure

The region of the *E* gene employed herein exhibited a 113 bp length, whereas the amplicons of the N1-N2 system of the *N* gene were 72 and 67 bp in length, respectively, all of which exhibited loop-bubble structures. Interestingly, 154 bp of the first half of the *ORF8* gene presented a loop-bubble structure, which was similar to that of the other two genes. Nevertheless, the third loop of the N1 system and the second loop of the *ORF8* gene were formed via four and seven canonical pairings, respectively. These structures can be considered scorpion-type primers or probes, which are often used in RT-qPCR; however, the loop-bubble structures used for these purposes are typically formed *via* 2–6 canonical pairings ( Figure 2). Table 1 summarizes the primer sequences used in this study.

**Figure 2.**
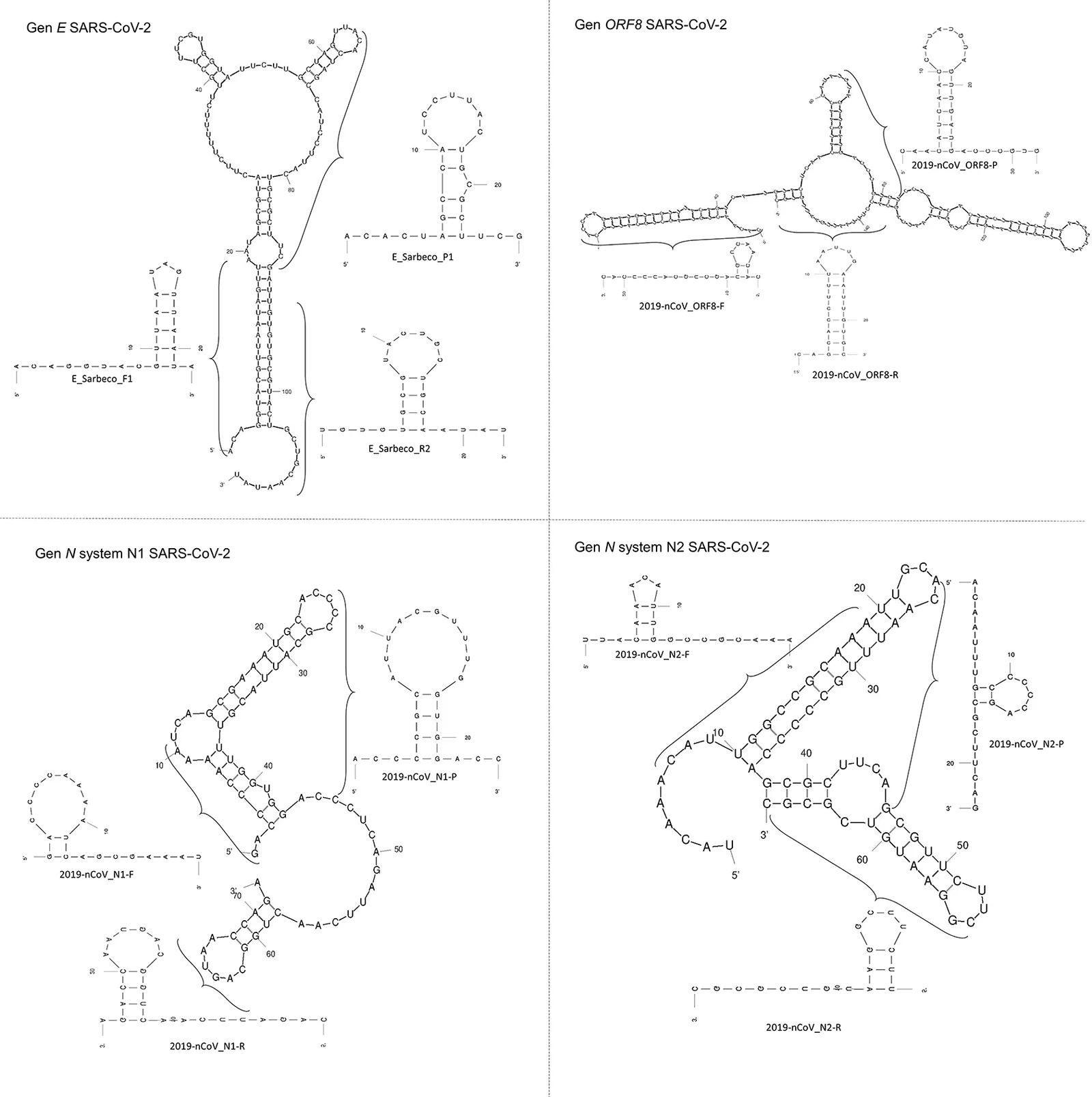
Secondary structures of the primers and probes used for genes *E*, *ORF8* and *N* (N1-N2). Stem and stem-bubble structures are observed in the 5' and 3' direction. The numbers represent the amount of nucleic acids used for each set and the parentheses individually delimit each primer and probe within each segment.

**Table 1.** RT-qPCR primer, probes, and substrate oligos designed in this study.

Gen	Type	Code	Sequence	Total nmole	Reference
*ORF8*	Primer Forward	FwO820CoV	CAYAACTGTAGCTGCATTTCAC	24.3	This work
	Primer Reverse	RvO820CoV	GCACAATTCAATTAAAGGTGCTG	21.9	
	Probe	TqMO820CoV	FAM-CAACATCAACCATATGTAGTTGATGACCCGTG-BBQ	8.7	
	Substrate Oligonucleotide	TrGtO8	CAYAACTGTAGCTGCATTTCACCAAGAATGTAGTTTACAG TCATGTACTCAACATCAACCATATGTAGTTGATGACCCGT GTCCTATTCACTTCTATTCTAAATGGTATATTAGAGTAGG AGCTAGAAAATCAGCACCTTTAATTGAATTGTGC	0.3	
*E*	Primer Forward	E_Sarbeco_F1	ACAGGTACGTTAATAGTTAATAGCGT	34.0	( Corman *et al.*, 2020)
	Primer Reverse	E_Sarbeco_R1	ATATTGCAGCAGTACGCACACA	49.8	
	Probe	E_Sarbeco_P1	FAM-ACACTAGCCATCCTTACTGCGCTTCG-BBQ	22.5	
*N*	Primer Forward	2019-nCoV_N1-F	GACCCCAAAATCAGCGAAAT	22.5	( Biotek, 2020)
	Primer Reverse	2019-nCoV_N1-R	TCTGGTTACTGCCAGTTGAATCTG	22.5	
	Probe	2019-nCoV_N1-P	FAM-ACCCCGCATTACGTTTGGTGGACC-BHQ	22.5	
	Primer Forward	2019-nCoV_N2-F	TTACAAACATTGGCCGCAAA	22.5	
	Primer Reverse	2019-nCoV_N2-R	GCGCGACATTCCGAAGAA	22.5	
	Probe	2019-nCoV_N2-P	FAM-ACAATTTGCCCCCAGCGCTTCAG-BHQ	22.5	
*RNase P*	Primer Forward	RP-F	AGATTTGGACCTGCGAGCG	54.3	( Corman *et al.*, 2020)
	Primer Reverse	RP-R	GAGCGGCTGTCTCCACAAGT	53.2	
	Probe	RP-P	FAM–TTCTGACCTGAAGGCTCTGCGCG–BHQ	12.8	

### SARS-CoV-2 detection *via E*, *ORF8*, and *N* RT-qPCR

A total of 49 samples were collected from October 25, 2020, to January 21, 2021, of which 22 tested positive and 27 were negative. The October samples were used to confirm that the *E*, *ORF8*, and *N* genes could be used as effective indicators to detect SARS-CoV-2 *via* RT-qPCR ( Table 2, Figure 3). However, given that our secondary structure and nucleotide ratio analyses indicated that the A:T ratio was always between 63% and 70% regardless of the consensus sequence, denaturing and dNTP solutions were also incorporated in these reactions (see the Methods for more details). These conditions generally improved the Ct values of the reactions compared to those of the commercial procedures; however, some exceptions were observed ( Table 2, Figure 3).

**Table 2.** RT-qPCR single gene SARS-CoV-2.

Sample	Collection/Process Date	Reagent	*E*	*ORF8*	N1	N2	*RNase P*	Sample	Collection/Process Date	Reagent	*E*	*ORF8*	N1	N2	*RNase P*
02	25/10/2020 18/11/2020	Comm	41.884	44.458	27.527	27.471	28.500	03	27/10/2020 19/11/2020	Comm	23.300	24.131	27.904	28.130	28.316
		Desn	39.498	59.736	28.837	28.598	28.763			Desn	24.518	24.539	27.489	28.017	28.900
		dNTPs	35.338	16.999	28.278	31.674	27.675			dNTPs	25.556	25.851	27.616	29.531	28.090
07	03/12/2020 04/12/2020	Comm	---	20.662	No determine	24.383	28.827	10	04/12/2020 17/12/2020	Comm	14.417	---	No determine	No determine	27.623
		Desn	36.554	23.382		24.195	30.331			Desn	21.025				27.188
11	05/12/2020 11/12/2020	Comm	20.806	19.893	No determine	---	32.268	12	06/12/2020 17/12/2020	Comm	29.291	26.454	No determine	---	27.776
		Desn	35.808	33.855		---	27.518			Desn	25.257	---		---	28.202
14	14/12/2020 19/12/2020	Comm	7.970	10.416 4.873	No determine	No determine	30.167	15	14/12/2020 20/12/2020	Comm	44.182	---	No determine	No determine	27.994
		Desn	21.082				31.051			Desn	---	---			25.933
16-21	14-18/12/2020 16-18/12/2020	Comm	Similar results to sample 15				+++	22	18/12/2020 27/12/2020	Comm	20.559	---	No determine	32.003	26.813
		Desn					+++			Desn	---	26.017		---	31.614
23	18/12/2020 27/12/2020	Comm	24.533	---	No determine	33.795	29.757	24	21/12/2020 27/12/2020	Comm	17.016	---	No determine	22.325	26.014
		Desn	---	25.500		---	34.371			Desn	---	17.192		31.312	31.392

*E*, *ORF8*, N1-N2 and *RNase P* = Primers and probes to WHO, this work, *N* gen from CDC and Universal control, respectively.

Values correspond to Ct result by RT-qPCR.

Dot line = Result not was generated.

Comm = Commercial reagent.

Desn = Denaturalization reagent.

dNTPs = dNTPs reagent.

+++ = Positive result.

**Figure 3.**
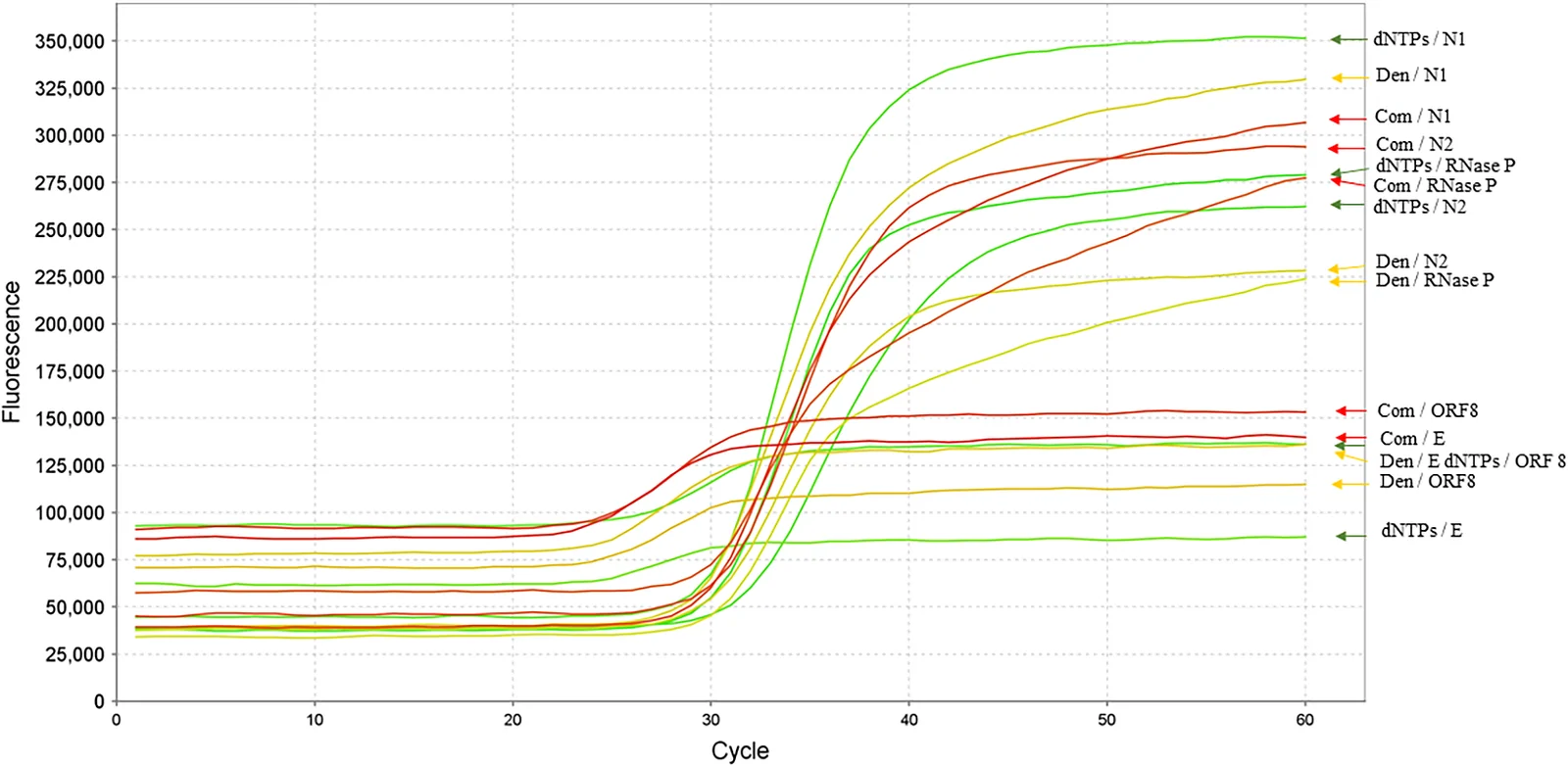
RT-qPCR curves for the detection of SARS-CoV-2 from patient-derived samples. RNA obtained from patients and primers and probes corresponding to the *E* gene (WHO), *ORF8* (this study), and the *N* gene (N1-N2; CDC). The green, yellow, and red arrows indicate the curves generated by denaturalization (Den), the dNTP reagent, and commercial kits, respectively. The *RNase P* gene was used as a control.

Given that all samples were freshly acquired and never stored, we concluded that the gradual loss of amplicon systems and adjuvant solutions began in December. This was confirmed first for the *E* gene system, then for the N2 system, and finally for *ORF8.* This trend was determined using ten samples collected from December 14 to 21, 2020. The results of our experiments could be generally classified into three outcomes depending on the viral detection system: (1) the system worked following the manufacturer’s instructions with the addition of the denaturing solution (2); the system rendered positive results with the denaturing solution but negative without it; and (3) the opposite of the second outcome ( Table 2).

### *In silico* estimation of *E*, *ORF8*, and *N* RT-PCR primer and probe combinations

Our analysis of the global SARS-CoV-2 mutation patterns based on the GenBank and GISAID databases indicated that most mutations began in March 2020, and subsequently increased significantly, relative to the SARS-CoV-2 reference genome (GenBank access: NC_045512.2). This trend was particularly noticeable for the quencher and forward primer sequences of the *ORF8* and *N* genes. Moreover, the potential primer and probe combinations for each gene increased substantially starting in November according to the GISAID database and were significantly higher in December. For example, the *ORF8* system and the N1 set exhibited 3.4×10 ^11^ and 1.7×10 ^12^ combinations, respectively, whereas the N2 set and *E* had only 1.4×10 ^5^ and 32 combinations ( Figure 1).

### Multiplex RT-qPCR

The multiplex RT-qPCR controls of systems *E*, *ORF8*, and *N* (N1) with the positive samples 02 and 03 rendered the expected results, which were thereafter confirmed using samples 05 and 06 obtained in October 2020. Nevertheless, using the same reaction conditions, the denaturing solution decreased the Ct values for the positive samples obtained in October but enhanced those of the samples obtained in November. In contrast, the dNTP solution had the opposite effect ( Table 3, Figure 4).

**Table 3.** RT-qPCR Multiplex to SARS-CoV-2.

Date	RT-PCR Multiplex	Reagent	Sample								
			02	03	05	06					
Collection			25/10/2020	27/10/2020	12/11/2020	09/11/2020					
Process			22/11/2020								
	*E*, *ORF8* and N1	Comm	27.377	18.290	33.299	35.341					
		Desn	27.109	18.050	35.149	35.919					
		dNTPs	28.057	18.845	32.792	34.256					
	*RNase P*	Comm	27.908	26.449	25.876	27.309					
			10	11	12	13	38	39			
Collection			04/12/2020	05/12/2020	06/12/2020	7/12/2020	15/01/2021	14/01/2021			
Process			18/01/2021								
	N1 and N2	Comm	19.961	30.558	28.461	36.556	24.581	---			
		Desn	19.219	27.389	27.947	---	23.230	23.961			
	*RNase P*	Comm	20.855	25.610	26.525	23.669	25.171	26.306			
		Desn	26.043	25.589	26.402	23.028	26.943	26.304			
			32	33	34						
Collection			26/12/2020	29/12/2020	29/12/2020						
Process			07/01/2021								
	*E*, *ORF8* and N1	Comm	---	---	---						
		Desn	31.194	36.784	35.523						
	*RNase P*	Comm	29.973	29.165	29.802						
		Desn	---	39.593	30.761						
			41	42	43	44	45	46	47	48	49
Collection			19/01/2021	21/01/2021	26/01/2021	24/01/2021	29/01/2021	29/01/2021	23/01/2021	23/01/2021	21/01/2021
Process			22/01/2021				30/01/2021				
	*E*, *ORF8* and N1	Comm	20.529	28.554	30.913	23.318	32.362	30.324	33.064	29.253	30.411
		Desn	26.471	24.536	30.160	21.325	30.444	31.857	28.459	29.515	28.695
	*RNase P*	Comm	29.098	24.786	23.353	23.130	28.733	25.742	24.343	24.664	26.368
		Desn	---	23.472	23.414	24.260	26.898	25.297	24.488	25.290	25.976

Nomenclature in Table 2.

**Figure 4.**
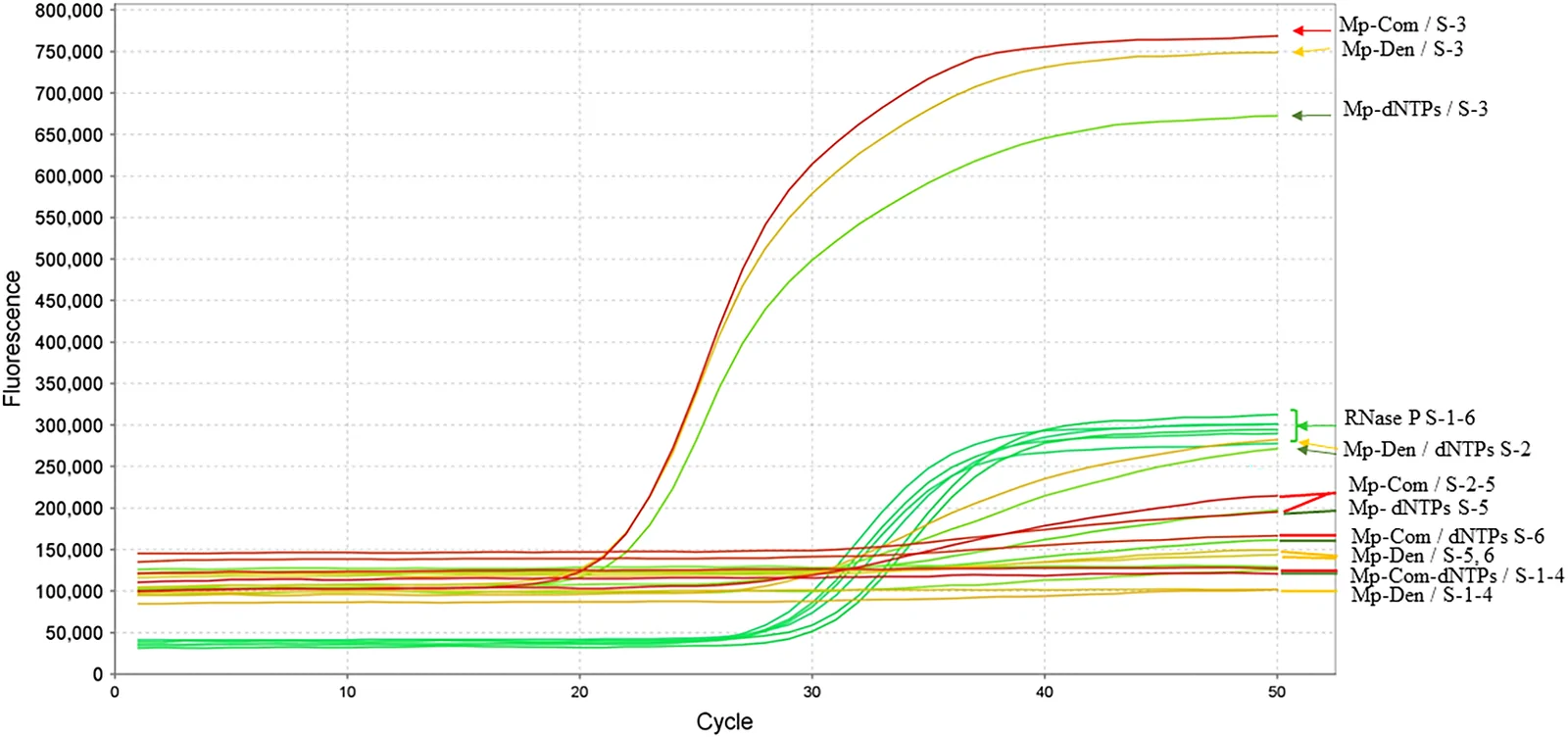
Multiplex RT-qPCR (Mp) curves for the detection of SARS-CoV-2 using *E*-, *ORF8*-, and *N* (N1)-specific. The RNA samples from 6 different patients are indicated with the respective numbers. For each curve, the first number in the nomenclature indicated the top result and the second the lower result. The *RNase P* gene was used as a control. The arrows and nomenclature are the same as in Figure 3.

The influence of the denaturing and dNTP solutions was further confirmed using nine samples obtained from December 4 to 7, 2020, and from January 14 to 15, 2021, using the N1–N2 primers and probe as described by the CDC. A total of six samples exhibited the originally documented pattern and the three remaining samples tested negative, both in terms of curve trends and Ct values ( Table 3, Figure 5). Therefore, the initial multiplex reaction effectively detected the SARS-CoV-2 virus in the nine samples obtained from January 19 to 29, 2021 ( Table 3).

**Figure 5.**
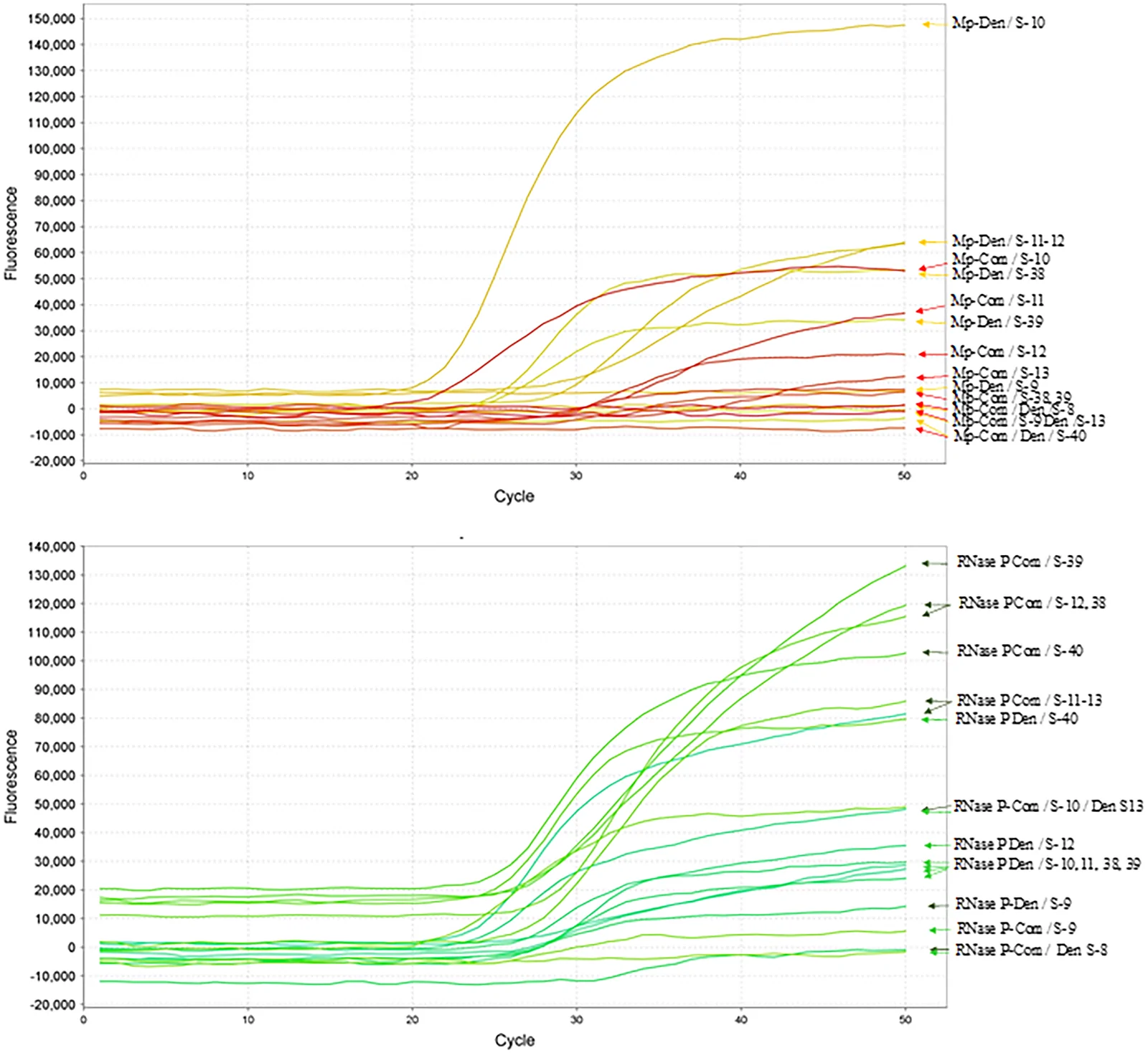
RT-qPCR Multiplex (Mp) curves for the detection of SARS-CoV-2 using from *N* gene (N1-N2). Primers and probes, as indicated by the CDC coupled with the denaturalization reagent. The RNA samples from 9 patients are indicated with the respective numbers. The multiplex curves corresponding to N1-N2 are indicated at the top, whereas the results corresponding to the *RNase P* primers and probe used as controls are indicated below. The abbreviators and nomenclature are the same as in Figure 1.

### Sensitivity, specificity and predictive values of SARS-CoV-2

The performance of single and multiplex RT-qPCRs with the solutions used showed that single denaturing was more sensitive than the commercial solution, but the latter is 33% more specific than the former. As for the multiplex reactions, the commercial solution showed non-significant difference in sensitivity with respect to the denaturing solution. Similarly, the specificity of the single reactions was better for the commercial solution with respect to the denaturing solution, but in the multiplex reactions this difference was not evident in the detection of true negatives as both solutions yielded results of 0. dNTPs were not evaluated due to the number of positive results ( Table 4).

**Table 4.** Sensitivity, specificity, PPV and NPV values for single and multiplex RT-qPCR reactions of SARS-CoV-2.

Calculation of sensitivity and specificity for single RT-qPCR					
Comm			Desn		
	Event	No Event		Event	No Event
Event	6	2	Event	2	8
No Event	8	1	No Event	0	0
Kappa:	-0.1333		Kappa:	0	
Mcnemar's Test P-Value:	0.1138		Mcnemar's Test P-Value:	0.01333	
Sensitivity:	0.4286		Sensitivity:	1.0	
Specificity:	0.3333		Specificity:	0.0	
Pos Pred Value (PPV) :	0.7500		Pos Pred Value (PPV) :	0.2	
Neg Pred Value (NPV) :	0.1111		Neg Pred Value (NPV) :	NaN	
Prevalence:	0.8235		Prevalence:	0.2	

Calculation of sensitivity and specificity for multiplex RT-qPCR					
Comm			Desn		
	Event	No Event		Event	No Event
Event	16	4	Event	15	2
No Event	2	0	No Event	5	0
Kappa:	-0.1379		Kappa:	-0.1493	
Mcnemar's Test P-Value:	0.6831		Mcnemar's Test P-Value:	0.4497	
Sensitivity:	0.8889		Sensitivity:	0.7500	
Specificity:	0.0000		Specificity:	0.0000	
Pos Pred Value (PPV) :	0.8000		Pos Pred Value (PPV) :	0.8824	
Neg Pred Value (NPV) :	0.0000		Neg Pred Value (NPV) :	0.0000	
Prevalence:	0.8182		Prevalence:	0.9091	

## Discussion

Since the first report of the SARS-CoV-2 virus and the beginning of the pandemic, the public health authorities indicated that the spread of this disease could be prevented by implementing rapid, cost-effective, and accurate diagnostic tools that would enable the analysis of large sample volumes across the globe. Many RT-qPCR-based diagnostic kits have since been developed based on SARS-CoV-2 marker genes ( Ruhan *et al.*, 2020; Nalla *et al.*, 2020). However, similar to the Berlin protocol ( Corman *et al.*, 2020), several of these kits require two or three consecutive reactions that must be conducted after the first results, thus prolonging the diagnosis process. Importantly, excessively long test procedures are not only an inconvenience but also pose a serious risk to patient health and promote disease dissemination.

To the best of our knowledge, our study is the first to demonstrate the use of three genes both independently or in multiplex reactions to detect the SARS-CoV-2 virus. This was achieved by combining the primers and probes for the *E* gene of the Berlin protocol, the *N* gene (N1-N2 set) of the CDC protocol, and the *ORF8* gene with reagents that promote the elimination of secondary structures and compensate for the variations in the nucleotide proportions of the SARS-CoV-2 genome. More importantly, these modifications enhance the SARS-CoV-2 detection efficiency of RT-qPCR, thus rendering results in as little as 12 hours.

These achievements were largely facilitated by our HPC analyses, which allowed for full SARS-CoV-2 genome alignments and database curation in a matter of minutes or hours using publicly available sequences from the GISAID and GenBank databases dating back to the first genomic characterization of the virus up until December 31, 2020 ( Zhu *et al.*, 2020). This allowed for the design and *in silico* validation of RT-qPCR primers and probes for viral detection. Nevertheless, even though our database consolidated genomic sequences from across the globe, it does not reflect the true distribution and frequency of viral variants by region, as the implementation of next-generation sequencing (NGS) technologies varies widely in different countries and regions depending on infrastructure and economic factors, thus creating biases in viral variant descriptions and genomic surveillance.

Given the aforementioned considerations, the consensus sequence-based primer and probe design conducted herein were also based on genome quality and quantity, as well as the computational capacity of our HPC cluster. This enabled the analysis of gene sequences in minutes or hours, thus allowing for the evolutionary and genomic characterization of the virus.

Another benefit of our global SARS-CoV-2 genome database is that it allowed for the prediction of secondary RNA and cDNA structures associated with the amplified region, thus providing insights into the final primer and probe nucleotide distributions and concentrations. This approach also enabled the specific formulation of denaturing solutions ( Kovarova & Draber, 2000) and nucleic acid synthesis to enhance RT-qPCR Ct values for different regions of the SARS-CoV-2 genome, as demonstrated in this study. All of these factors facilitate the validation of RT-qPCR-based assays and substantially decrease the likelihood of false-negative results.

The reagents and methods described herein allow for an immediate, facile, and cost-effective detection of the SARS-CoV-2 virus; which was demonstrated by comparing the Cts of the positive results used to determine sensitivity and specificity with those published for other countries ( Arakawa *et al.,* 2024; Aranha *et al.*, 2021; Chen *et al.*, 2022). Nevertheless, the proposed method still relied on the use of two or three SARS-CoV-2 genes in addition to the *RNase P* universal control in the same RT-qPCR plate to ensure viral detection accuracy. Additionally, the high mutation rates of this virus will inevitably increase detection costs and decrease test accuracy over time, as demonstrated with the December 2020 samples. Therefore, the SARS-CoV-2 genome databases must be continually updated to preserve the accuracy and applicability of our proposed procedure.

Based on our findings, we concluded that multiplex RT-qPCR is an optimal solution for the aforementioned limitations, even though the Cts used to establish sensitivity and specificity did not show a difference in some samples between commercial methods compared to the one proposed here. However, different fluorescent dyes were required to differentiate the different probes, thus increasing operating costs associated not only with the dyes themselves but also the acquisition of multiple-channel thermocyclers to detect different fluorescent signals. Despite these disadvantages, combining the detection of the *E*, *ORF8*, and *N* genes increases the likelihood of identifying a fluorescent signal, making SARS-CoV-2 detection more robust and reliable. In this sense, the timely and accurate detection of the virus would allow caregivers to promptly implement pertinent measures and prevent disease progression and transmission. Moreover, the RT-qPCR procedures proposed herein are highly modular, allowing health professionals to select only the gene-specific primers and probes that they deem necessary for different diagnostic needs.

Another advantage of multiplex RT-qPCR assays is that they enable the analysis of multiple samples from a single patient ( Grobe *et al.*, 2021). Specifically, preliminary studies have demonstrated that robust diagnoses can be achieved using four samples obtained at different dates. However, this requires additional monitoring (e.g., clinical records) to optimize pool design. Taken together, our findings indicate that integrating HPC, probe and primer design, denaturing and dNTP solutions, and single and multiplex RT-qPCR protocols will contribute to the timely detection of the SARS-CoV-2 virus, thus minimizing its spread.

## Data Availability

This genomic sequence is available in GenBank: GenBank: Severe acute respiratory syndrome coronavirus 2 isolate Wuhan-Hu-1, complete genome. Accession number NC_045512; https://www.ncbi.nlm.nih.gov/nuccore/NC_045512. GitHub: Steps to download and align genomic sequences from GenBank in the GUANE-1 supercomputer from High Performance and Scientific Computing Center of the Industrial University of Santander (SC3UIS). This software is also used for manually downloaded sequences such as GISAID. For both databases or others, the script eliminates the undetermined sequences indicated with “n” within the genome or study sequence. https://github.com/druedaplata/bio Zenodo: SARS-CoV-2 genomic data obtained for this study are available in: Denaturing and dNTPs reagents improve SARS-CoV-2 detection via single and multiplex RT-qPCR. https://doi.org/10.5281/zenodo.6337537 ( Cadena-Caballero *et al.*, 2022). This project contains the following extended data:
- SARS-CoV-2 genomic sequences from the GenBank and GISAID databases and their alignments. Data are available under the terms of the Creative Commons Zero “No rights reserved” data waiver (CC0 1.0 Public domain dedication).
