# Denaturing and dNTPs reagents improve SARS-CoV-2 detection *via* single and multiplex RT-qPCR

**DOI:** 10.12688/f1000research.109673.3

**Published:** 2026-05-02

**Authors:** Cristian E. Cadena-Caballero, Lina M. Vera-Cala, Carlos Barrios-Hernandez, Diego Rueda-Plata, Lizeth J. Forero-Buitrago, Carolina S. Torres-Jimenez, Erika Lizarazo-Gutierrez, Mayra Agudelo-Rodriguez, Francisco Martinez-Perez

**Affiliations:** 1 Grupo de Investigación Computo Avanzado y a Gran Escala - CAGE, Universidad Industrial de Santander, Bucaramanga, Santander, 680006, Colombia; 2 Grupo de Investigación en Demografía, Salud Pública y Sistemas de Salud - GUINDESS, Universidad Industrial de Santander, Bucaramanga, Santander, 680006, Colombia; 3 Centro de Supercomputación y Cálculo Científico de la Universidad Industrial de Santander -SC3UIS, Universidad Industrial de Santander, Bucaramanga, Santander, 680006, Colombia

**Keywords:** SARS-CoV-2, Diagnosis RT-qPCR, Adjuvant formulation, Primer and probe design, High performance computing

## Abstract

**Background:**

The COVID-19 pandemic, caused by SARS-CoV-2, highlighted the need for accurate and scalable diagnostic tools such as RT-qPCR. However, false-negative results may occur due to viral mutations and RNA secondary structures within target regions.

**Methods:**

High-performance computing (HPC) was used to compile SARS-CoV-2 genomic sequences from GenBank and GISAID and generate consensus sequences for primer and probe design. A region within the *ORF8* gene was selected and evaluated alongside targets from the *E* and *N* genes and the *RNase P* control. Nasopharyngeal swab samples were collected from patients with a prior clinical diagnosis consistent with SARS-CoV-2 infection, as well as from volunteers, and total RNA was extracted using the MagMAX kit. RT-qPCR assays were performed in both single and multiplex formats. Denaturing solutions composed of tetraethylammonium chloride and dimethyl sulfoxide, as well as adjusted dNTP proportions based on viral nucleotide composition, were evaluated. Exploratory Ct-based performance metrics were estimated using predefined threshold criteria with the Caret package in R.

**Results:**

A total of 126,576 SARS-CoV-2 genomes collected between January and December 2020 were used to construct a reference dataset. A target region within the *ORF8* gene exhibiting predicted secondary structures was selected for primer and probe design. Forty-nine clinical samples were analyzed, of which 22 tested positive across the evaluated gene targets. Variability in detection patterns was observed across sampling periods. The evaluated formulations were associated with changes in Ct values in both single and multiplex RT-qPCR assays, depending on the conditions and sample set.

**Conclusions:**

The incorporation of denaturing solutions and the adjustment of nucleotide proportions were associated with changes in RT-qPCR performance under the evaluated experimental conditions. These findings suggest that RNA secondary structure and nucleotide composition may influence assay behavior; however, further studies are required to assess the broader applicability of this approach.

## Introduction

On January 23, 2020, the SARS-CoV-2 virus ( *Coronaviridae*: *Betacoronavirus*: *Severe acute respiratory syndrome-related coronavirus*) was declared a public health emergency by the World Health Organization (WHO) International Health Regulations (IHR) Emergency Committee. At the time, the global public health authorities established that the spread of SARS-CoV-2 could be prevented if every nation adopted solid strategies for rapid and accurate disease detection ( WHO, 2020).

Diagnostic methods based on gene-specific primers and probes for the detection of viruses *via* gene amplification include quantitative real-time polymerase chain reaction (RT-qPCR) or reverse transcription loop-mediated isothermal amplification (RT-LAMP), both of which can be conducted using oropharyngeal and nasopharyngeal swab samples from patients ( Kevadiya *et al.*, 2021). Among these, the former method is considered the most sensitive and accurate for the detection of SARS-CoV-2 and other viruses ( Martín *et al.*, 2021). This procedure could be effectively implemented due to the characterization of the viral genome, which encompasses approximately 10 genes ( Zhu *et al.*, 2020). Therefore, WHO authorized the Berlin protocol which relies on the following genes: *ORF1ab*, which encodes proteins that enable viral replication ( Nur *et al.*, 2015); *Spike* ( *S*), which interacts with the receptor of the host’s angiotensin-converting enzyme 2 (ACE2) ( Wan *et al.*, 2020); and *E*, which encodes the structural envelop protein ( Tahan *et al.*, 2021). Other protocols such as the 2019-nCoV TaqMan RT-qPCR Kit authorized by the United States Centers for Disease Control and Prevention (CDC) utilize two regions of the *N* gene (N1-N2), which encode the nucleocapsid phosphoprotein ( Navarathna *et al.*, 2021). Moreover, both protocols use the *RNase P* gene as a control to assess the efficiency of the RT-qPCR protocol ( WHO, 2009).

Nevertheless, RT-qPCR-based diagnosis is conducted on a per-gene basis and therefore its widespread implementation would be both impractical and costly. Therefore, multiplex RT-qPCR ( *e.g.*, the CDC kit or combined quantification of the *ORF1ab* and *S* genes) could be implemented as a promising approach to meet the current demand for accurate and cost-efficient diagnosis ( Kudo *et al.*, 2020). Sample pooling is another approach that could increase population-wide SARS-CoV-2 diagnosis rates and has therefore been approved and implemented in several countries ( Grobe *et al.*, 2021). However, sample numbers and sampling structure may limit the implementation of this strategy ( Grobe *et al.*, 2021).

Although RT-qPCR-based SARS-CoV-2 diagnostic tools are generally considered the gold standard for disease detection, several studies have determined that this approach is prone to return false-negative results due to gene mutations (which is often the case for the *E* and *N* genes) ( Hasan *et al.*, 2021; Tahan *et al.*, 2021) or primer dimer formation ( Jaeger *et al.*, 2021). Another factor that affects RT-qPCR efficiency is the secondary structure of the RNA to be characterized/quantified ( Hammerling *et al.*, 2020).

Previous studies have demonstrated the role of RNA secondary structure in viral evolution and transcript regulation ( Andrews *et al.*, 2021; Huston *et al.*, 2021; Rangan *et al.*, 2020; Wacker *et al.*, 2020). However, their potential impact on RT-qPCR performance in clinical samples remains insufficiently characterized. Furthermore, the effects of adjuvants such as dimethyl sulfoxide (DMSO) or ammonium salts on RT-qPCR efficiency are not fully understood ( Kovarova & Draber, 2000).

In this context, we used High-Performance Computing (HPC) to compile a global dataset of SARS-CoV-2 genomes available up to December 2020 and selected a region of the *ORF8* gene for evaluation in both single and multiplex RT-qPCR assays, alongside targets from the *E* and *N* genes. Additionally, we assessed the potential impact of adjusting denaturing solution composition and nucleotide proportions on RT-qPCR performance.

This study was designed as an exploratory and methodological approach to evaluate how RNA secondary structure and nucleotide composition may influence RT-qPCR assay performance under defined experimental conditions. The implications of these findings are discussed in the following sections.

## Methods

### Ethics statement

This study was approved by the Research Ethics Committees of the Industrial University of Santander, the Chicamocha Clinic, and the Chicamocha Clinical Laboratory (L3C) (Bucaramanga, Santander, Colombia). The study participants were hospitalized patients diagnosed with SARS-CoV-2, individuals presenting to the emergency room, or volunteers. All participants provided written informed consent and voluntarily participated in our study. Further, all participants were kept anonymous and were informed that the present study was conducted strictly for research purposes and that its outcomes were not intended to serve as treatment or diagnosis.

### SARS-CoV-2 sample collection and storage

Nasopharyngeal swab samples were acquired by L3C personnel. Two samples were obtained per study participant, one for clinical diagnosis, as authorized by the Ministry of Health and Social Protection of Colombia, and the other for our study. The samples were placed in Universal Transport Medium (UTM) and stored at −80°C until required for downstream analyses ( Rogers *et al.*, 2020). Samples were collected over a one-week period and packaged in accordance with applicable Colombian health regulations ( Ministry of Health and Social Protection, 2020) prior to transport and processing. All samples were transferred to the Central Research Laboratory of the Faculty of Health Sciences at the Industrial University of Santander (LCI-FS-UIS) and maintained at 4°C during handling and processing. In all cases, written informed consent was obtained prior to sample collection, and documentation was properly verified and archived in accordance with institutional and national ethical guidelines.

Because sample collection was not conducted under a predefined experimental sampling framework but was instead constrained by voluntary participation and ethical requirements during the COVID-19 pandemic, an anonymized coding system was implemented to ensure participant confidentiality, in accordance with Resolution 8430 of 1993 and the applicable Colombian ethical framework ( Ministry of Health and Social Protection, 1993). The alphanumeric code consisted of two digits corresponding to the participant, followed by “CO” (Colombia) and a consecutive number assigned to each sample.

Subsequently, samples were aliquoted in a Class II, Type A2 biosafety cabinet (Thermo Fisher Scientific). A total of three aliquots were obtained per sample, including one 200 μL aliquot used immediately for total RNA extraction and two 650 μL backup aliquots stored at −80°C.

### SARS-CoV-2 genome database consolidation

To account for reported mutations in SARS-CoV-2 genomes that could affect primer and probe binding and potentially generate false-negative results, a region encoding the accessory protein ORF8 was selected for analysis. This region was chosen based on its predicted mutation profile, and monthly consensus sequences were generated to assess its identity pattern across newly available genomes. Publicly available SARS-CoV-2 genome sequences were obtained from the GenBank and GISAID databases (RRID:SCR_002760; RRID:SCR_018251) ( Sayers *et al.*, 2021; Shu & McCauley, 2017). To gather data from the GenBank database, a Python script (RRID:SCR_008394) was executed in the GUANE-1 High Performance and Scientific Computing Center of the Industrial University of Santander (SC3UIS) through the Entrez Programming Utilities interface using the Biopython package (RRID:SCR_013249; RRID:SCR_007173) ( Cock *et al.*, 2009; Geer *et al.*, 2010), whereas all GISAID data were manually downloaded. Genomes with uncharacterized regions/nucleotides were discarded using another Python script (RRID:SCR_008394), which was used to conduct monthly FASTA sequence alignments using the MAFFT software (RRID:SCR_011811) ( Katoh & Standley, 2013) coupled with previously described DNA loss model parameters ( Martínez-Pérez *et al.*, 2002, 2007). The consensus sequences were generated using BioEdit version 7.2 (RRID:SCR_007361) with a 100% threshold frequency ( Hall, 1999).

### Design and synthesis of *ORF8*-specific primers, probes, and substrate oligos

Using the SARS-CoV-2 (NC_045512) reference genome and consensus sequences from January to April 2020 ( Zhu *et al.*, 2020), an approximately 150-base pair (bp) region of the *ORF8* gene was selected based on the secondary structure of its RNA transcript, which in turn was predicted using the algorithms proposed by Zuker ( Zuker & Jacobson, 1998) via the Mfold software (RRID:SCR_001360) ( Zuker, 2003). Thus, the last set containing the codons of the central region of the *ORF8* gene, which encodes the secretion protein ORF8, was chosen because it allows proper viral adhesion to the host cell ( Chan *et al.*, 2020). The obtained sequence was used as a template to create primers, a TaqMan FAM-BBQ probe, and substrate oligos for RT-qPCR. The specificity of the aforementioned molecules was confirmed via GenBank BLAST analyses (RRID:SCR_004870) ( Ye *et al.*, 2006). Genes *E* and *N* from the Berlin and CDC protocols were used as controls ( Biotek, 2020; Corman *et al.*, 2020).

All molecules were synthesized by Bioneer (Korea). The primers were purified *via* separation on a reverse-phase cartridge, whereas the probe and substrate oligos were purified *via* high-performance liquid chromatography (HPLC) and polyacrylamide gel electrophoresis (PAGE), respectively. The *ORF8* RT-qPCR conditions were implemented as described by the Berlin protocol ( Corman *et al.*, 2020) using the aforementioned molecules coupled with the 2019-nCoV TaqMan RT-qPCR Kit (Norgen Biotek Corp) developed by the CDC ( Biotek, 2020).

### Preparation of denaturing solutions and adjustment of dNTP concentrations

A:T and G:C ratios were calculated based on the monthly SARS-CoV-2 consensus sequences to obtain an average for each nucleotide. These averages were then used to determine the minimum concentrations of TEA (ABCAM-USA), DMSO (Scharlab-Spain), and dNTPs (100 mM each, Promega-USA) in molecular-grade ultrapure water (Promega-USA), in addition to the MgSO _4_ concentration recommended by the Berlin protocol.

### RNA extraction

Total RNA extraction was conducted using the MagMAX™ Viral/Pathogen II (MVP II) Nucleic Acid Isolation Kit (2000 RXNs) (Applied Biosystems-USA) using a KingFisher Duo Prime (5400110) DNA/RNA extraction system according to the manufacturer’s instructions (Thermo Fisher Scientific-USA) ( Fang *et al.*, 2007).

### SARS-CoV-2 single and multiplex RT-qPCR

RT-qPCR was conducted using the *ORF8*-specific primers and probe designed herein, in addition to the *E* (Berlin protocol) and *N* genes (N1-N2; CDC protocol). The *RNase P* gene was used as an external control, as proposed by both of the aforementioned protocols. The reactions were conducted using the SuperScript III One-Step RT-qPCR System with Platinum Taq DNA Polymerase (Ref. 11732088; Invitrogen-USA) and the 2019-nCoV TaqMan RT-qPCR Kit (Ref. TM67100; Norgen-Biotek-Canada). Each reaction for each diagnostic system was conducted in either 15- or 25-μL reaction volumes consisting of 2 μL of patient-derived purified RNA, 2× One-Step RT-qPCR Master Mix, 2× nuclease-free buffer, and the respective primers/probe at the concentrations recommended by the CDC. Moreover, a denaturing stock solution was added to obtain a final concentration of 0.7% TEA, 0.2% DMSO, and 0.8 mMol MgSO _4_. The dNTP reagent had a final concentration of 12 mMol dATP-dTTP, 10 mMol dCTP-dGTP, and 0.8 mMol MgSO _4_. The reaction conditions were the following: 55°C for 15 minutes, 95°C for 3 minutes followed by 45 cycles of 95°C for 15 s and 58°C for 30 s for the SuperScript™ III One-Step RT-qPCR kit; and 95°C for 3 s followed by 55°C for 20 s for the 2019-nCoV TaqMan RT-qPCR kit. Fluorescence signals were quantified using a QuantStudio 1 Real-Time PCR System (No. A40427) in a 96-well 0.2 μL block (Thermo Fisher Scientific-USA).

The above-described procedure was conducted using two different Multiplex One-Step RT-qPCR protocols. In the first instance, the primers and probes for the *E*, *ORF8*, and *N* (N1) genes were mixed, whereas the other reaction was performed by mixing the N1 and N2 sets of the *N* gene. The *RNase P* gene was independently assessed in both cases. All reactions were performed as described above.

### Exploratory Ct-based performance analysis of SARS-CoV-2 RT-qPCR assays

Single and multiplex RT-qPCR assays were compared based on Ct values obtained under identical experimental conditions. Comparative analyses were performed at both the individual primer/probe level and at the sample level, allowing evaluation of performance differences between assay formats.

To estimate sensitivity, specificity, Positive Predictive Value (PPV), and Negative Predictive Value (NPV) for the single and multiplex RT-qPCR assays, a custom script was implemented in R using the Caret package ( https://github.com/GenomicUIS/Sensitivity-specificity-PPV-and-NPV-for-SARS-CoV-2.git) ( Kuhn, 2008; R Core Team, 2020). All scripts and processed datasets used in these analyses are publicly available in the referenced repository.

Because this study was designed as an exploratory methodological study and not as a clinical diagnostic validation, performance estimates were derived from predefined Ct-based operational criteria applied uniformly across assays. For individual reactions, Ct values between 18 and 35 were considered within the operational positive range. Ct values outside this interval, including values >35 or <18, were conservatively classified as non-positive or indeterminate for this exploratory internal analysis.

Ct values were subsequently pooled and evaluated at the sample level. A sample was considered positive when at least one primer/probe set met the predefined operational Ct criterion. For multiplex RT-qPCR assays, the same Ct-based criteria were applied, but results were evaluated collectively at the sample level.

These metrics were used to compare assay behavior under the evaluated experimental conditions. Because no independent external clinical validation was available, and because access to additional clinical information was restricted by ethical and legal considerations, these estimates should not be interpreted as measures of absolute diagnostic accuracy.

## Results

### SARS-CoV-2 GenBank and GISAID databases

A total of 19,317 genomes were retrieved from the GenBank database from January to October 2020 based on our search criteria, whereas 107,259 complete genomes were obtained from the GISAID database between January and December 2020. In both datasets, the number of available sequences increased progressively over time. However, the frequency of base substitutions in the monthly consensus sequences for the *E*, *ORF8*, and *N* genes was higher in the GISAID dataset compared to GenBank ( Figure 1).

**Figure 1.**
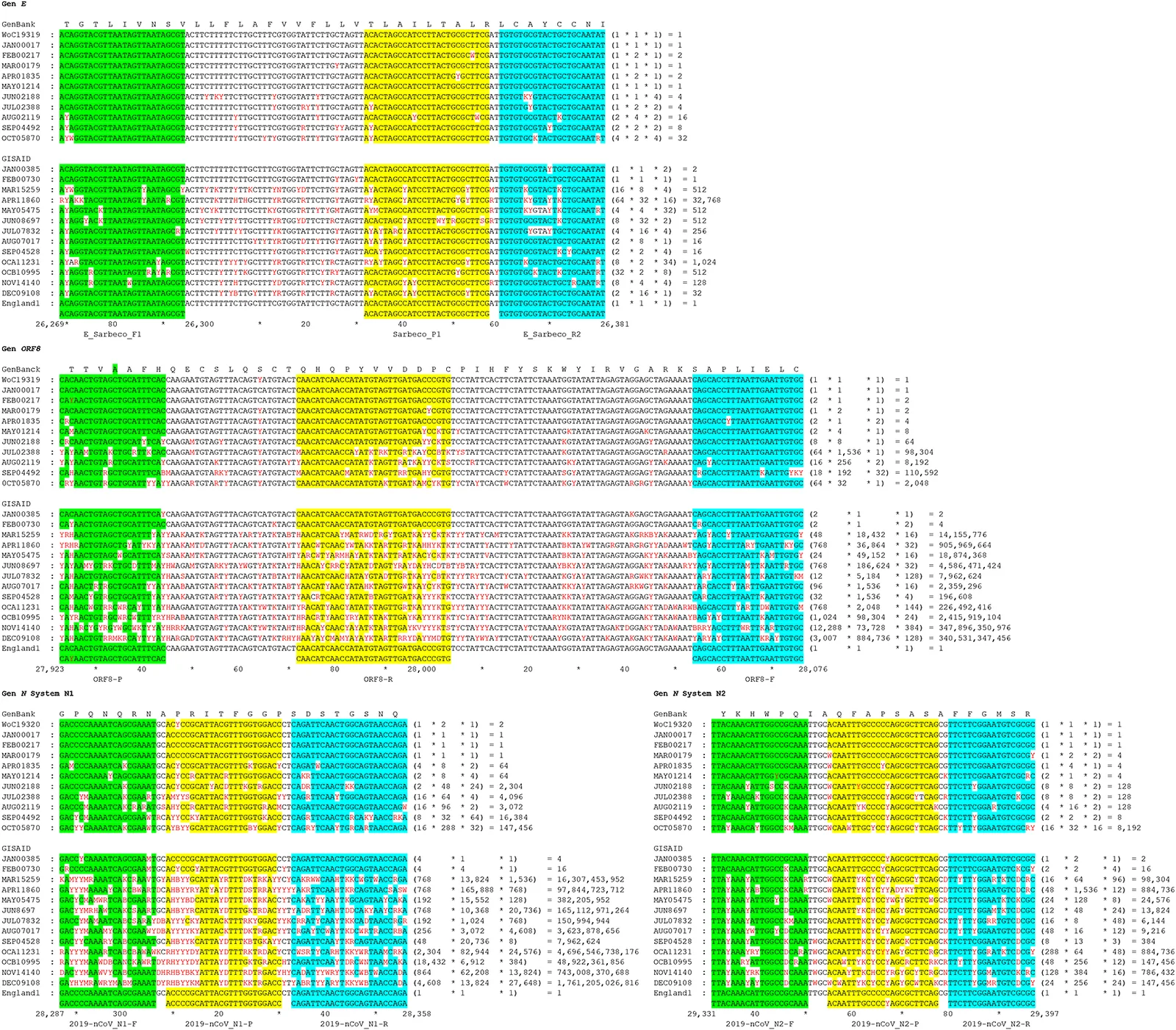
Primers and probes used for RT-qPCR detection of SARS-CoV-2. Nucleotide sequences are indicated as follows: forward primers are shown in green boxes, reverse primers in blue boxes, and probes in yellow boxes. Conceptual translation is displayed above each alignment. Numbers indicate nucleotide position within the sequence, and nucleotide combinations at each position follow international nomenclature. The nucleotide positions relative to the SARS-CoV-2 reference genome are shown in the lower consensus alignment.

### Primer, probe, and substrate oligonucleotides design for SARS-CoV-2 detection based on RNA secondary structure

The region of the *E* gene employed herein exhibited a 113 bp length, whereas the amplicons of the N1-N2 system of the *N* gene were 72 and 67 bp in length, respectively. All regions exhibited loop-bubble structures. A 154 bp region within the first half of the *ORF8* gene also presented a loop-bubble structure similar to those observed in the *E* and *N* genes. The third loop of the N1 system and the second loop of the *ORF8* gene were formed by four and seven canonical base pairings, respectively. These structures are comparable to scorpion-type primer or probe configurations used in RT-qPCR; however, such loop-bubble structures are typically formed by 2–6 canonical base pairings ( Figure 2). Table 1 summarizes the primer sequences used in this study.

**Figure 2.**
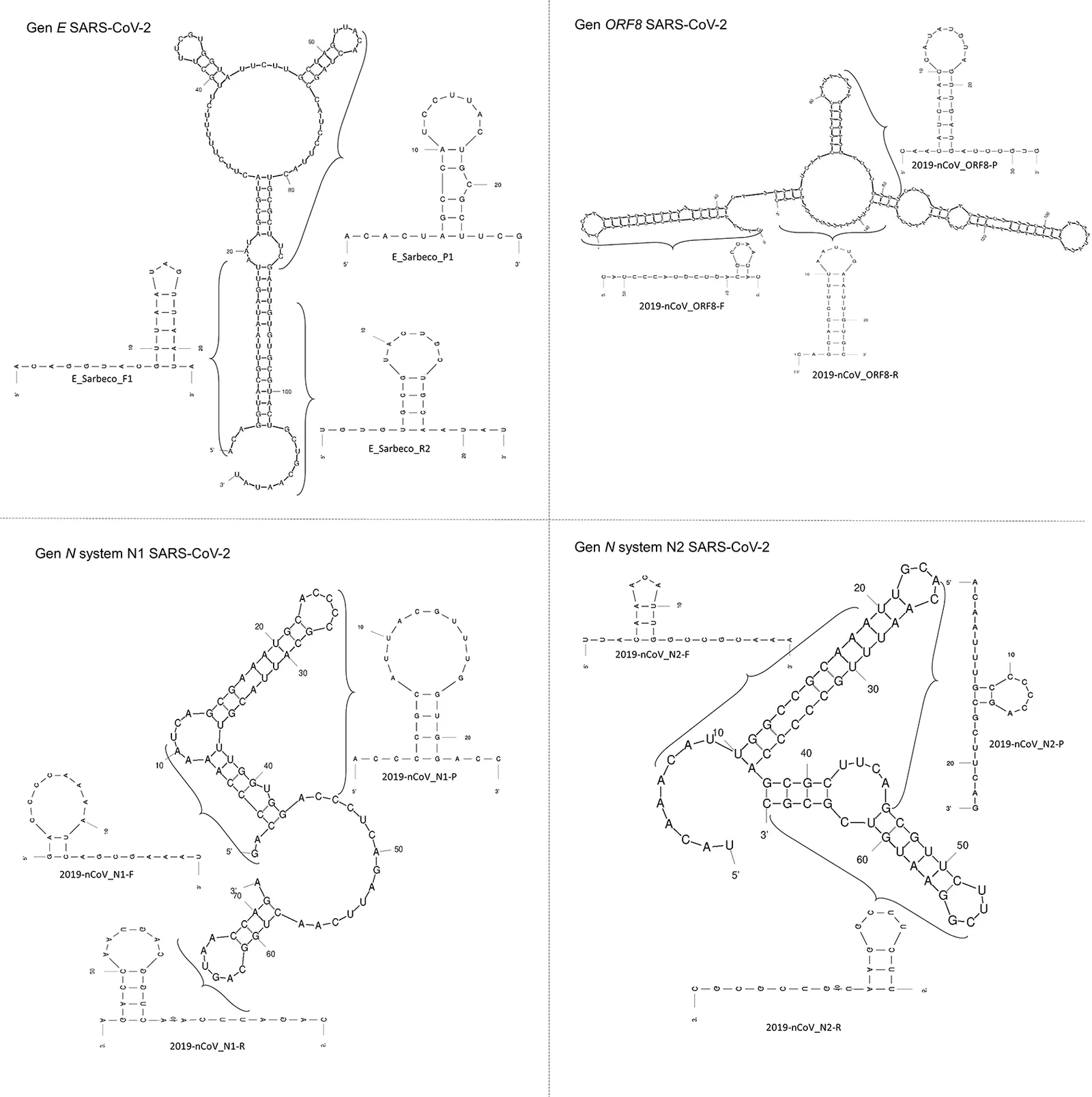
Predicted secondary structures of primers and probes used for the *E*, *ORF8*, and *N* (N1–N2) genes. Stem and stem–loop structures are observed in both the 5′ and 3′ orientations. Numbers indicate the number of nucleotides in each structure, and parentheses delineate individual primers and probes within each segment.

**Table 1.** Primers, probes, and substrate oligonucleotides used for RT-qPCR in this study.

Gen	Type	Code	Sequence	Total nmole	Reference
*ORF8*	Primer Forward	FwO820CoV	CAYAACTGTAGCTGCATTTCAC	24.3	This work
	Primer Reverse	RvO820CoV	GCACAATTCAATTAAAGGTGCTG	21.9	
	Probe	TqMO820CoV	FAM-CAACATCAACCATATGTAGTTGATGACCCGTG-BBQ	8.7	
	Substrate Oligonucleotide	TrGtO8	CAYAACTGTAGCTGCATTTCACCAAGAATGTAGTTTACAG TCATGTACTCAACATCAACCATATGTAGTTGATGACCCGT GTCCTATTCACTTCTATTCTAAATGGTATATTAGAGTAGG AGCTAGAAAATCAGCACCTTTAATTGAATTGTGC	0.3	
*E*	Primer Forward	E_Sarbeco_F1	ACAGGTACGTTAATAGTTAATAGCGT	34.0	( Corman *et al.*, 2020)
	Primer Reverse	E_Sarbeco_R1	ATATTGCAGCAGTACGCACACA	49.8	
	Probe	E_Sarbeco_P1	FAM-ACACTAGCCATCCTTACTGCGCTTCG-BBQ	22.5	
*N*	Primer Forward	2019-nCoV_N1-F	GACCCCAAAATCAGCGAAAT	22.5	( Biotek, 2020)
	Primer Reverse	2019-nCoV_N1-R	TCTGGTTACTGCCAGTTGAATCTG	22.5	
	Probe	2019-nCoV_N1-P	FAM-ACCCCGCATTACGTTTGGTGGACC-BHQ	22.5	
	Primer Forward	2019-nCoV_N2-F	TTACAAACATTGGCCGCAAA	22.5	
	Primer Reverse	2019-nCoV_N2-R	GCGCGACATTCCGAAGAA	22.5	
	Probe	2019-nCoV_N2-P	FAM-ACAATTTGCCCCCAGCGCTTCAG-BHQ	22.5	
*RNase P*	Primer Forward	RP-F	AGATTTGGACCTGCGAGCG	54.3	( Corman *et al.*, 2020)
	Primer Reverse	RP-R	GAGCGGCTGTCTCCACAAGT	53.2	
	Probe	RP-P	FAM–TTCTGACCTGAAGGCTCTGCGCG–BHQ	12.8	

### SARS-CoV-2 detection *via E*, *ORF8*, and *N* RT-qPCR

A total of 49 samples were collected between October 25, 2020, and January 21, 2021, of which 22 tested positive and 27 tested negative. Samples collected in October were used to evaluate the detection of SARS-CoV-2 using the *E*, *ORF8*, and *N* gene targets by RT-qPCR ( Table 2, Figure 3). Based on the secondary structure and nucleotide composition analyses, which indicated an A:T ratio between 63% and 70% across consensus sequences, denaturing and dNTP solutions were incorporated into the reactions (see Methods for details). Under these conditions, changes in Ct values were observed compared to the commercial procedures, although variability was detected among samples ( Table 2, Figure 3).

**Table 2.** Single-target RT-qPCR results for SARS-CoV-2 detection.

Sample	Collection/Process Date	Reagent	*E*	*ORF8*	N1	N2	*RNase P*	Sample	Collection/Process Date	Reagent	*E*	*ORF8*	N1	N2	* RNase P*
02	25/10/2020 18/11/2020	Comm	41.884	44.458	27.527	27.471	28.500	03	27/10/2020 19/11/2020	Comm	23.300	24.131	27.904	28.130	28.316
		Desn	39.498	59.736	28.837	28.598	28.763			Desn	24.518	24.539	27.489	28.017	28.900
		dNTPs	35.338	16.999	28.278	31.674	27.675			dNTPs	25.556	25.851	27.616	29.531	28.090
07	03/12/2020 04/12/2020	Comm	---	20.662	Not determined	24.383	28.827	10	04/12/2020 17/12/2020	Comm	14.417	---	Not determined	Not determined	27.623
		Desn	36.554	23.382		24.195	30.331			Desn	21.025				27.188
11	05/12/2020 11/12/2020	Comm	20.806	19.893	Not determined	---	32.268	12	06/12/2020 17/12/2020	Comm	29.291	26.454	Not determined	---	27.776
		Desn	35.808	33.855		---	27.518			Desn	25.257	---		---	28.202
14	14/12/2020 19/12/2020	Comm	7.970	10.416 4.873	Not determined	Not determined	30.167	15	14/12/2020 20/12/2020	Comm	44.182	---	Not determined	Not determined	27.994
		Desn	21.082				31.051			Desn	---	---			25.933
16-21	14-18/12/2020 16-18/12/2020	Comm	Similar results to sample 15				+++	22	18/12/2020 27/12/2020	Comm	20.559	---	Not determined	32.003	26.813
		Desn					+++			Desn	---	26.017		---	31.614
23	18/12/2020 27/12/2020	Comm	24.533	---	Not determined	33.795	29.757	24	21/12/2020 27/12/2020	Comm	17.016	---	Not determined	22.325	26.014
		Desn	---	25.500		---	34.371			Desn	---	17.192		31.312	31.392

*E*, *ORF8*, N1-N2 and *RNase P* = Primers and probes to WHO, this work, *N* gen from CDC and Universal control, respectively.

Values correspond to Ct result by RT-qPCR.

Dot line = Result not was generated.

Comm = Commercial reagent.

Desn = Denaturalization reagent.

dNTPs = dNTPs reagent.

+++ = Positive result.

**Figure 3.**
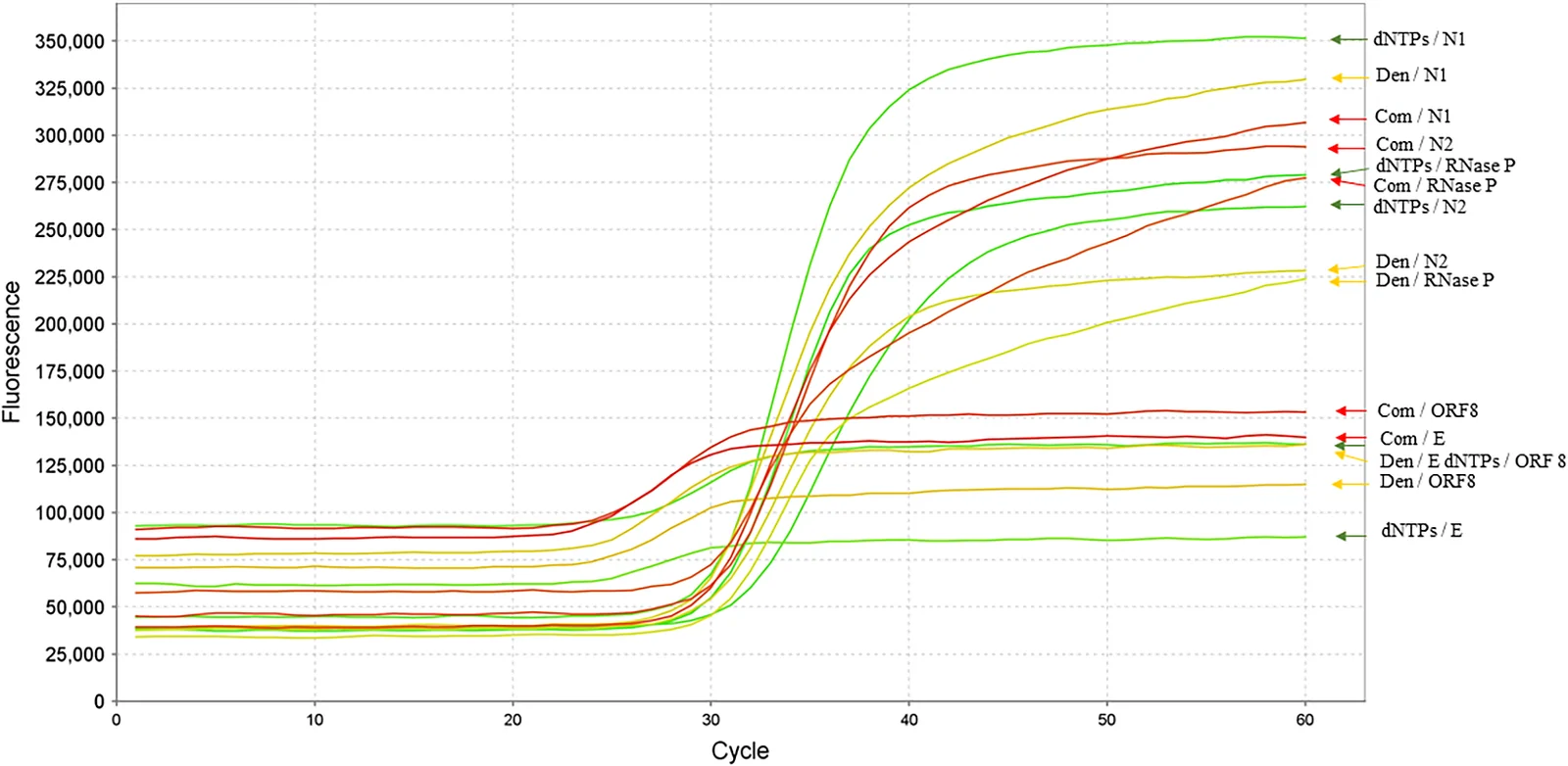
RT-qPCR amplification curves for SARS-CoV-2 detection in patient-derived samples. RNA extracted from patient samples was analyzed using primers and probes targeting the *E* gene (WHO protocol), *ORF8* (this study), and the *N* gene (N1–N2; CDC protocol). Green, yellow, and red arrows indicate amplification curves obtained using denaturing solution (Den), dNTP-adjusted conditions, and commercial kits, respectively. The *RNase P* gene was used as an internal control.

A decrease in amplification performance was observed in samples collected from December onward. This effect was first detected in the *E* gene system, followed by the N2 system and subsequently *ORF8*. This pattern was evaluated using ten samples collected between December 14 and 21, 2020. The observed results were classified into three outcomes depending on the detection system: (1) positive results were obtained following the manufacturer’s protocol with the addition of the denaturing solution; (2) positive results were obtained only in the presence of the denaturing solution; and (3) positive results were obtained only in the absence of the denaturing solution ( Table 2).

### *In silico* estimation of *E*, *ORF8*, and *N* RT-qPCR primer and probe combinations

Analysis of global SARS-CoV-2 mutation patterns using GenBank and GISAID databases showed that the number of mutations increased from March 2020 onward relative to the reference genome (GenBank accession: NC_045512.2). This trend was particularly evident in the quencher and forward primer regions of the *ORF8* and *N* genes. The number of potential primer and probe combinations also increased over time, with a marked rise observed from November onward in the GISAID dataset. Higher values were observed in December. For example, the *ORF8* system and the N1 set exhibited 3.4×10 ^11^ and 1.7×10 ^12^ possible combinations, respectively, whereas the N2 set and the *E* gene exhibited 1.4×10 ^5^ and 32 combinations ( Figure 1).

### Multiplex RT-qPCR

Multiplex RT-qPCR controls using the *E*, *ORF8*, and *N* (N1) systems with positive samples 02 and 03 produced amplification profiles consistent with detection. Similar patterns were observed in samples 05 and 06 collected in October 2020. Under the same reaction conditions, the addition of the denaturing solution was associated with decreased Ct values in samples collected in October, whereas increased Ct values were observed in samples collected in November. In contrast, the dNTP solution exhibited an opposite trend ( Table 3, Figure 4).

**Table 3.** Multiplex RT-qPCR results for SARS-CoV-2 detection.

Date	RT-qPCR Multiplex	Reagent	Sample								
			02	03	05	06					
Collection			25/10/2020	27/10/2020	12/11/2020	09/11/2020					
Process			22/11/2020								
	*E*, *ORF8* and N1	Comm	27.377	18.290	33.299	35.341					
		Desn	27.109	18.050	35.149	35.919					
		dNTPs	28.057	18.845	32.792	34.256					
	*RNase P*	Comm	27.908	26.449	25.876	27.309					
			10	11	12	13	38	39			
Collection			04/12/2020	05/12/2020	06/12/2020	7/12/2020	15/01/2021	14/01/2021			
Process			18/01/2021								
	N1 and N2	Comm	19.961	30.558	28.461	36.556	24.581	---			
		Desn	19.219	27.389	27.947	---	23.230	23.961			
	*RNase P*	Comm	20.855	25.610	26.525	23.669	25.171	26.306			
		Desn	26.043	25.589	26.402	23.028	26.943	26.304			
			32	33	34						
Collection			26/12/2020	29/12/2020	29/12/2020						
Process			07/01/2021								
	*E*, *ORF8* and N1	Comm	---	---	---						
		Desn	31.194	36.784	35.523						
	*RNase P*	Comm	29.973	29.165	29.802						
		Desn	---	39.593	30.761						
			41	42	43	44	45	46	47	48	49
Collection			19/01/2021	21/01/2021	26/01/2021	24/01/2021	29/01/2021	29/01/2021	23/01/2021	23/01/2021	21/01/2021
Process			22/01/2021				30/01/2021				
	*E*, *ORF8* and N1	Comm	20.529	28.554	30.913	23.318	32.362	30.324	33.064	29.253	30.411
		Desn	26.471	24.536	30.160	21.325	30.444	31.857	28.459	29.515	28.695
	*RNase P*	Comm	29.098	24.786	23.353	23.130	28.733	25.742	24.343	24.664	26.368
		Desn	---	23.472	23.414	24.260	26.898	25.297	24.488	25.290	25.976

Nomenclature in Table 2.

**Figure 4.**
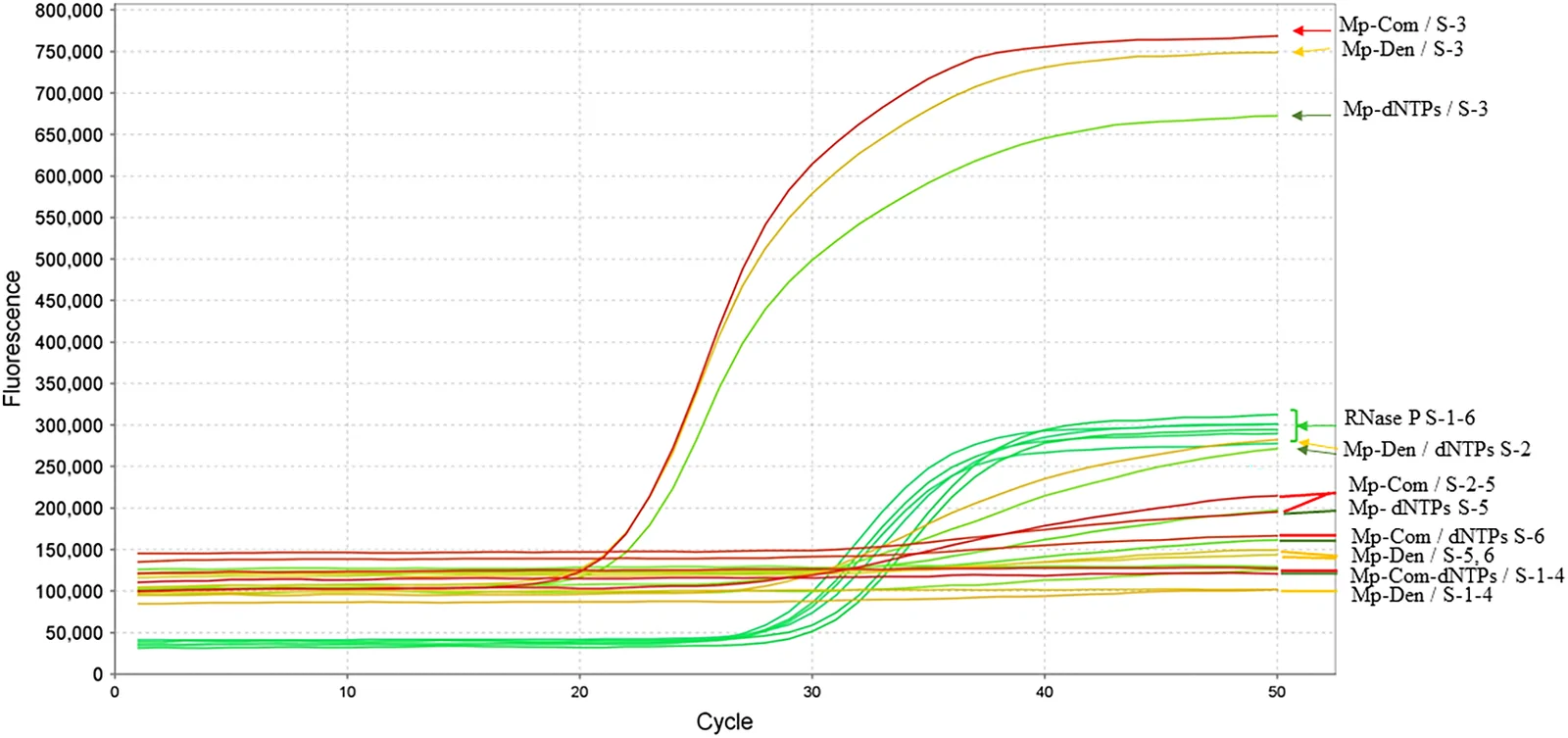
Multiplex RT-qPCR (Mp) amplification curves for SARS-CoV-2 detection using *E*, *ORF8*, and *N* (N1) targets. RNA samples from six patients are indicated by their corresponding identifiers. For each curve, the first number in the nomenclature corresponds to the upper panel, and the second to the lower panel. The *RNase P* gene was used as an internal control. Arrows and nomenclature are consistent with those described in Figure 3.

The influence of the denaturing and dNTP solutions was further evaluated using nine samples collected between December 4 and 7, 2020, and January 14 and 15, 2021, using the N1–N2 primers and probe described by the CDC. Six samples exhibited the same pattern described above, whereas three samples tested negative based on both amplification curves and Ct values ( Table 3, Figure 5). The multiplex RT-qPCR assay detected SARS-CoV-2 in samples collected between January 19 and 29, 2021 ( Table 3).

**Figure 5.**
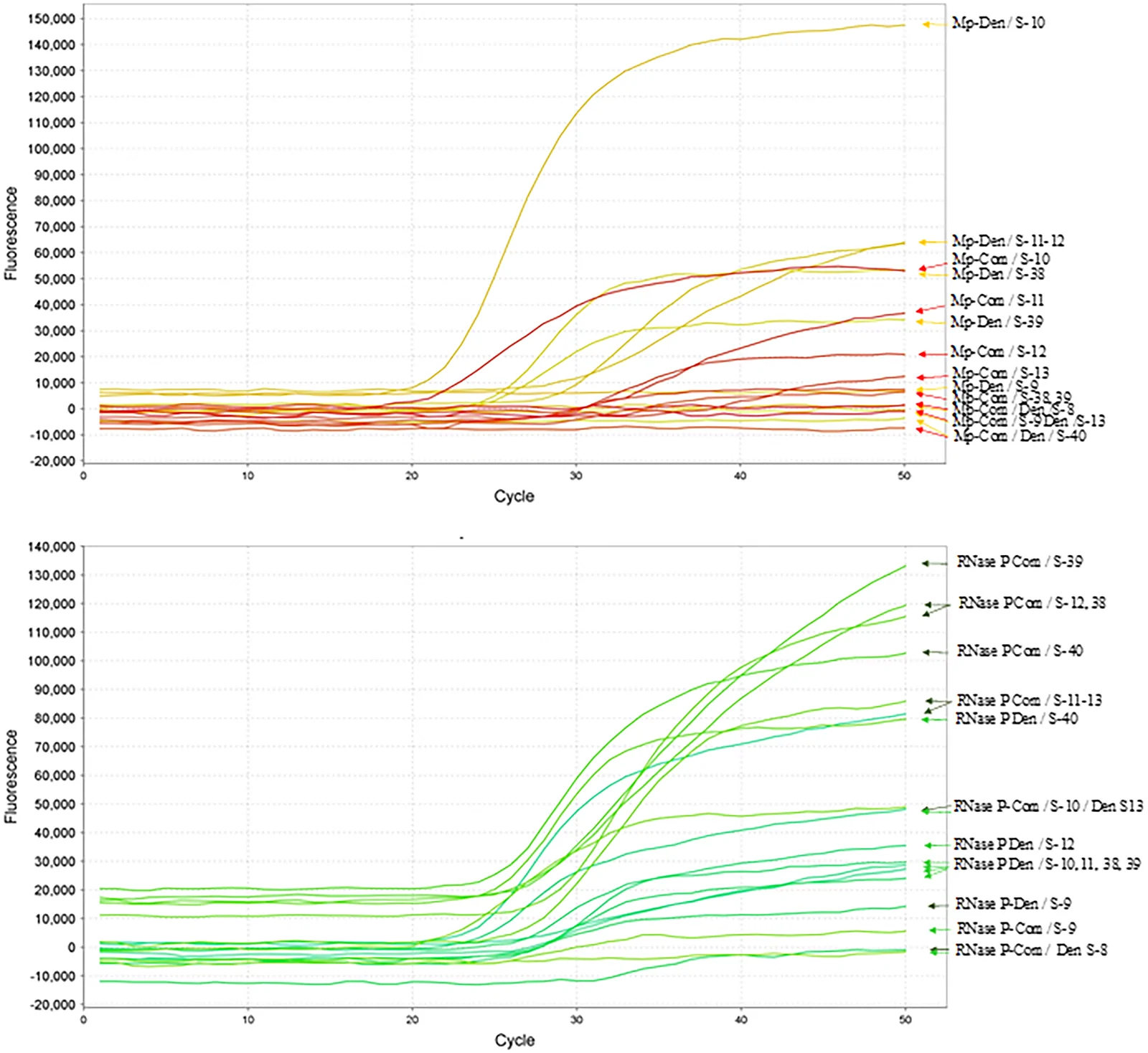
RT-qPCR Multiplex (Mp) curves for the detection of SARS-CoV-2 using the *N* gene (N1-N2). CDC-described primers and probes were used in combination with the denaturing reagent. The RNA samples from 9 patients are indicated with the respective numbers. The multiplex curves corresponding to N1-N2 are indicated at the top, whereas the results corresponding to the *RNase P* primers and probe used as controls are indicated below. The abbreviations and nomenclature are the same as in Figure 3.

### Sensitivity, specificity, and predictive values of SARS-CoV-2 detection

Under the predefined Ct-based classification framework, the denaturing solution was associated with higher apparent sensitivity values than the commercial solution in single RT-qPCR assays, whereas the commercial solution showed higher apparent specificity values. In multiplex RT-qPCR assays, no major differences in apparent sensitivity were observed between commercial and denaturing conditions.

In multiplex reactions, specificity values were 0.0 under both evaluated conditions in this dataset. Therefore, these metrics should be interpreted as exploratory descriptors of assay behavior under the defined experimental conditions rather than as evidence of clinical diagnostic performance. The dNTP conditions were not included in this analysis because the number of evaluable positive results was limited ( Table 4).

**Table 4.** Exploratory Ct-based performance metrics for single-target and multiplex RT-qPCR assays under different reagent conditions.

Calculation of sensitivity and specificity for single RT-qPCR					
Comm			Desn		
	Event	No Event		Event	No Event
Event	6	2	Event	2	8
No Event	8	1	No Event	0	0
Kappa:	-0.1333		Kappa:	0	
Mcnemar's Test P-Value:	0.1138		Mcnemar's Test P-Value:	0.01333	
Sensitivity:	0.4286		Sensitivity:	1.0	
Specificity:	0.3333		Specificity:	0.0	
Pos Pred Value (PPV) :	0.7500		Pos Pred Value (PPV) :	0.2	
Neg Pred Value (NPV) :	0.1111		Neg Pred Value (NPV) :	NaN	
Prevalence:	0.8235		Prevalence:	0.2	

Calculation of sensitivity and specificity for multiplex RT-qPCR					
Comm			Desn		
	Event	No Event		Event	No Event
Event	16	4	Event	15	2
No Event	2	0	No Event	5	0
Kappa:	-0.1379		Kappa:	-0.1493	
Mcnemar's Test P-Value:	0.6831		Mcnemar's Test P-Value:	0.4497	
Sensitivity:	0.8889		Sensitivity:	0.7500	
Specificity:	0.0000		Specificity:	0.0000	
Pos Pred Value (PPV) :	0.8000		Pos Pred Value (PPV) :	0.8824	
Neg Pred Value (NPV) :	0.0000		Neg Pred Value (NPV) :	0.0000	
Prevalence:	0.8182		Prevalence:	0.9091	

Performance metrics were calculated using predefined Ct-based operational criteria for internal comparative analysis and should not be interpreted as externally validated diagnostic accuracy estimates.

## Discussion

Since the first report of the SARS-CoV-2 and the onset of the pandemic, public health strategies have emphasized the need for rapid, cost-effective, and accurate diagnostic tools capable of processing large numbers of samples. Numerous RT-qPCR-based diagnostic systems have been developed targeting SARS-CoV-2 marker genes ( Ruhan *et al.*, 2020; Nalla *et al.*, 2020). However, similar to the Berlin protocol ( Corman *et al.*, 2020), several of these approaches require sequential reactions, which may prolong diagnostic workflows and limit efficiency.

To our knowledge, this study represents one of the first approaches to evaluate the use of three gene targets both independently and in multiplex RT-qPCR reactions for SARS-CoV-2 detection. This strategy combined primers and probes from the *E* gene (Berlin protocol), the *N* gene (CDC protocol), and the *ORF8* gene, together with reagents designed to reduce RNA secondary structures and account for nucleotide composition variability. These modifications were associated with changes in Ct values under the evaluated experimental conditions.

High-Performance Computing (HPC) analyses enabled large-scale SARS-CoV-2 genome alignment and database curation using publicly available sequences from GISAID and GenBank up to December 31, 2020 ( Zhu *et al.*, 2020). This approach supported primer and probe design as well as *in silico* evaluation. However, the compiled database may not fully represent the global distribution of viral variants, as sequencing capacity varies across regions.

The use of a global genomic dataset also enabled the prediction of RNA and cDNA secondary structures associated with amplified regions, informing primer and probe design. Additionally, this approach supported the formulation of denaturing solutions and nucleotide compositions that were associated with changes in Ct values under the evaluated conditions ( Kovarova & Draber, 2000). These factors may contribute to differences in RT-qPCR assay performance within the experimental framework of this study.

The reagents and methods evaluated showed Ct values comparable to those reported in previous studies ( Arakawa *et al.,* 2024; Aranha *et al.*, 2021; Chen *et al.*, 2022). However, the proposed approach relies on the use of multiple gene targets and internal controls to ensure detection accuracy. Furthermore, the high mutation rate of SARS-CoV-2 may affect assay performance over time, highlighting the need for continuous updates of genomic databases.

Based on the results obtained, multiplex RT-qPCR represents a potential approach to address some of the limitations described. However, no consistent differences in Ct values were observed across all samples when compared with commercial methods. Additionally, the implementation of multiplex assays requires multiple fluorescent dyes and specialized equipment, which may increase operational costs. Despite these considerations, combining multiple gene targets may increase the likelihood of detecting a signal under specific conditions. The modular nature of the proposed approach allows flexibility in selecting gene targets according to diagnostic requirements.

Another advantage of multiplex RT-qPCR is the possibility of analyzing multiple samples from a single patient ( Grobe *et al.*, 2021). Although preliminary studies suggest that sampling at different time points may improve detection consistency, this approach requires additional clinical monitoring to optimize its implementation.

Taken together, these findings suggest that integrating HPC-based design, primer and probe optimization, and RT-qPCR protocols may support improvements in SARS-CoV-2 detection workflows under the evaluated conditions.

### Limitations

This study has several limitations. The sample size was constrained by logistical factors and low participation rates during the pandemic, including reluctance to undergo additional sampling procedures ( McElfish *et al*., 2021), as well as compliance with legal and ethical requirements. Consequently, only participants who provided informed consent were included.

Additionally, sample classification was based on Ct threshold criteria without independent external validation. Therefore, the reported performance metrics reflect comparative assay behavior under the evaluated conditions rather than absolute diagnostic accuracy.

These limitations should be considered when interpreting the results. Further studies incorporating larger datasets and additional validation approaches are required to assess the broader applicability of the proposed methodology. In addition, clinical characterization of participants, including symptom severity and extent of infection, was not available for analysis because this information was protected under the informed consent framework and applicable legal restrictions.

## Conclusions

The results of this study suggest that the incorporation of denaturing solutions and the adjustment of nucleotide proportions may influence RT-qPCR performance under the evaluated experimental conditions. These effects were observed in both single and multiplex assays, although variability was detected across samples.

The integration of computational approaches, including HPC-based genome analysis, with primer and probe design provided a framework to evaluate the potential impact of RNA secondary structure and nucleotide composition on assay performance.

Given the exploratory nature of this study and the limited sample size, these findings should be interpreted within the context of the experimental design. Additional studies incorporating larger datasets and independent validation strategies will be necessary to determine the broader applicability of the proposed approach.

## Data Availability

This genomic sequence is available in GenBank: GenBank: Severe acute respiratory syndrome coronavirus 2 isolate Wuhan-Hu-1, complete genome. Accession number NC_045512; https://www.ncbi.nlm.nih.gov/nuccore/NC_045512. All genomic data used in this study were obtained from publicly available databases, including GenBank and GISAID. The sequences analyzed, along with their corresponding alignments, are available in the associated Zenodo repository: Zenodo: Denaturing and dNTPs reagents improve SARS-CoV-2 detection *via* single and multiplex RT-qPCR. https://doi.org/10.5281/zenodo.6337537 ( Cadena-Caballero *et al*., 2022). This repository contains:
• SARS-CoV-2 genomic sequences obtained from GenBank and GISAID databases. • Multiple sequence alignments used for primer and probe design. Additionally, all scripts used for sequence processing and analysis are publicly available on GitHub:
•
https://github.com/druedaplata/bio. •
https://github.com/GenomicUIS/Sensitivity-specificity-PPV-and-NPV-for-SARS-CoV-2 These scripts were used for downloading, filtering, and aligning genomic sequences, including the removal of undetermined nucleotides (“N”) from the datasets, as well as for statistical analysis. All data and resources are openly accessible and available under the terms of the Creative Commons Zero (CC0 1.0) Public Domain Dedication, ensuring full transparency and reproducibility of the analyses performed in this study.
