# Supplementary material for: Denaturing and dNTPs reagents improve SARS-CoV-2 detection via single and multiplex RT-qPCR
Source: F1000Res. 2026 May 2;11:331. Originally published 2022 Mar 18. [Version 3] doi: 10.12688/f1000research.109673.3 (PMC13638059; doi:10.12688/f1000research.109673.3)
Supplement: Peer review history

# Reviewer report: Reviewer response for version 3

**DOI:** 10.5256/f1000research.198314.r500887

**Published:** 2026-07-17

**Authors:** Lina Silva-Trujillo

**Affiliations:** 1 Oswaldo Cruz Institute, Rio de Janeiro, Brazil

This manuscript investigates whether modifications to RT-qPCR reaction conditions, specifically the addition of denaturing agents (TEA and DMSO) and the adjustment of dNTP concentrations based on viral nucleotide composition, can improve SARS-CoV-2 detection in singleplex and multiplex assays. The study addresses an interesting technical question and presents an alternative strategy for optimizing molecular diagnostic assays. Overall, the manuscript is clearly written, well organized, and cites relevant literature. The experimental approach is appropriate, and the results support the overall conclusions. I have a few suggestions that could further improve the clarity and reproducibility of the work.

 The section describing the preparation of the denaturing solutions and the adjustment of dNTP concentrations could benefit from additional methodological detail. The authors indicate that nucleotide frequencies were calculated from monthly SARS-CoV-2 consensus sequences, but it would be helpful to specify how many genomes were analyzed, which database or software was used, and how these values were translated into the final dNTP concentrations. Likewise, providing a brief explanation of how the TEA and DMSO concentrations were selected would improve the reproducibility of the proposed protocol.

 Related to this point, I would appreciate a slightly expanded discussion on the rationale for adjusting the dNTP composition according to the nucleotide content of the viral genome. Since RT-qPCR amplifies relatively short target regions, it would be interesting to explain why the nucleotide composition of the complete viral genome was chosen as the basis for this optimization strategy. A brief discussion of the underlying rationale would strengthen this aspect of the manuscript.

 I also found Table 3 somewhat difficult to interpret. The denaturing solution appeared to reduce Ct values in samples collected during October but increase Ct values in samples collected during November, whereas the adjusted dNTP solution showed the opposite trend. These observations are interesting, and I think the manuscript would benefit from a brief discussion of possible explanations, such as differences in viral load, RNA quality, circulating variants, or other experimental factors. If available, presenting ΔCt values or measures of variability could also help readers interpret these findings.

 Regarding Table 4, I would appreciate additional clarification on how sensitivity, specificity, positive predictive value, and negative predictive value were calculated. In particular, the reported specificity of 0.0 for the multiplex assays is somewhat unexpected and would benefit from further explanation. If feasible, including the contingency tables or briefly describing the calculations would make these results easier to interpret. Given the limited number of clinical samples included in the study, it may also be helpful to emphasize that these performance estimates should be considered exploratory.

 Finally, although the oligonucleotides were commercially synthesized and additional quality control experiments are not necessarily expected, including the purity specifications provided by the manufacturer, together with the complete probe chemistry (fluorophore, quencher, and any additional modifications), would further improve the reproducibility of the methodology.

 Overall, I found this to be an interesting and well-conducted study addressing a relevant topic in molecular diagnostics. The proposed strategy has potential practical value, and the manuscript is clearly presented. I believe that addressing the suggestions above would further strengthen the work and improve its value for readers interested in RT-qPCR assay optimization.

Is the work clearly and accurately presented and does it cite the current literature?

Yes

If applicable, is the statistical analysis and its interpretation appropriate?

Partly

Are all the source data underlying the results available to ensure full reproducibility?

Yes

Is the study design appropriate and is the work technically sound?

Partly

Are the conclusions drawn adequately supported by the results?

Yes

Are sufficient details of methods and analysis provided to allow replication by others?

Partly

Reviewer Expertise:

Virology, antiviral drug discovery, molecular virology, RT-qPCR assay development and optimization, virus-host interactions, cell biology, molecular biology, antiviral screening, and emerging viral diseases.

I confirm that I have read this submission and believe that I have an appropriate level of expertise to confirm that it is of an acceptable scientific standard.

# Response

**Published:** 2026-08-01

**Authors:** Francisco Martinez-Perez

**Affiliations:** School of Biology, Industrial University of Santander, Bucaramanga, Santander, Colombia

**Dear Reviewer 3,**

 We appreciate your valuable comments and wish to inform you that all points have been fully addressed.

 Regarding the number of genomes analyzed, the total count and the source databases are mentioned in the first paragraph of the Results section. To assist the reader, we have included a link to the GitHub repository, where the process is described in detail. Additionally, at the end of that paragraph, we indicate the calculation used to obtain the total number of genomes.

 Furthermore, we modified the paragraph titled " *Preparation of denaturing solutions and adjustment of dNTP concentrations*" to provide greater clarity regarding the volumes used in the solutions, while maintaining the concentrations described in the " *SARS-CoV-2 single and multiplex RT-qPCR*" section. Likewise, in the Discussion section, we explain the rationale for choosing the nucleotide composition of the viral genome, and to reinforce this concept, we have included the citation for Bong et al. (2024), which has been added to the manuscript's reference section.

 Additionally, as the reviewer pointed out, to help readers interpret these findings, we have added the ΔCt values corresponding to the denaturing and dNTP conditions to Table 3, normalized against the commercial control.

 In this new version, we have also updated the repository containing the R scripts designed to determine specificity and sensitivity, including detailed usage information to facilitate the reproducibility of the methodology for readers. The link is: https://github.com/GenomicUIS/Sensitivity-specificity-PPV-and-NPV-for-SARS-CoV-2.git.

 Finally, Table 1 has been modified to include the purification specifications for the primers/probes used in the RT-qPCR, as provided by the manufacturer, as well as the complete chemical composition of the probes (including the fluorophore, quencher, and additional modifications). Information not reported by the manufacturer is identified as "NR".

 We remain available for any further observations and thank you again for your time and dedication in reviewing our work.

# Reviewer report: Reviewer response for version 3

**DOI:** 10.5256/f1000research.198314.r481060

**Published:** 2026-05-05

**Authors:** Juan Sebastian Quintero Barbosa

**Affiliations:** 1 University of Virginia, Charlottesville, Virginia, USA

The authors have addressed my comments

Is the work clearly and accurately presented and does it cite the current literature?

Partly

If applicable, is the statistical analysis and its interpretation appropriate?

Partly

Are all the source data underlying the results available to ensure full reproducibility?

Partly

Is the study design appropriate and is the work technically sound?

Partly

Are the conclusions drawn adequately supported by the results?

Partly

Are sufficient details of methods and analysis provided to allow replication by others?

Partly

Reviewer Expertise:

Virology, immunology, vaccine research, molecular biology, and experimental assay evaluation. My assessment is primarily focused on study design, methodological clarity, data interpretation, and whether the conclusions are adequately supported by the results.

I confirm that I have read this submission and believe that I have an appropriate level of expertise to confirm that it is of an acceptable scientific standard.

# Reviewer report: Reviewer response for version 2

**DOI:** 10.5256/f1000research.163834.r469433

**Published:** 2026-03-28

**Authors:** Juan Sebastian Quintero Barbosa

**Affiliations:** 1 University of Virginia, Charlottesville, Virginia, USA

This manuscript presents an interesting exploratory study on the optimization of SARS-CoV-2 RT-qPCR detection through in silico design of an ORF8-targeted system and the evaluation of modified reaction conditions in both single and multiplex assays. The topic is relevant, and the integration of computational design with experimental testing in clinical samples is a clear strength of the work. Overall, I believe the study has value as a methodological contribution, but several aspects would benefit from clarification and a more cautious interpretation. For this reason, I responded “Partly” to all six evaluation questions.

 For the question of whether the work is clearly and accurately presented and cites the current literature, I selected Partly because the manuscript is generally understandable and addresses a relevant body of literature, but some statements are stronger than the data support and some sections would benefit from clearer wording and greater precision.

 For the question of whether the study design is appropriate and the work technically sound, I selected Partly because the overall concept is appropriate and the study includes both in silico and experimental components, but the validation is relatively limited and does not fully support the strength of some of the claims made by the authors.

 For the question of whether sufficient details of methods and analysis are provided to allow replication, I selected Partly because the manuscript includes useful methodological information, but some aspects still require clarification, particularly how sample-level classifications were assigned and how the different assay formats were compared.

 For the question of whether the statistical analysis and its interpretation are appropriate, I selected Partly because the authors provide performance metrics and comparative analyses, but the interpretation should be more cautious. In particular, the basis for sensitivity, specificity, and related measures should be explained more clearly, and their limitations should be acknowledged more explicitly.

 For the question of whether all source data underlying the results are available to ensure full reproducibility, I selected Partly because the manuscript makes an effort toward transparency, but I was not fully convinced that all underlying information needed for complete reproducibility is presented in a sufficiently clear and accessible manner.

 For the question of whether the conclusions are adequately supported by the results, I selected Partly because the study provides promising findings, but the conclusions regarding improved diagnostic performance, especially in multiplex RT-qPCR, seem somewhat broader than what the current dataset can firmly support.

 In summary, I consider this a useful exploratory study with methodological interest, but I recommend clearer presentation of the methods, more cautious interpretation of the performance analyses, and moderation of the conclusions.

Is the work clearly and accurately presented and does it cite the current literature?

Partly

If applicable, is the statistical analysis and its interpretation appropriate?

Partly

Are all the source data underlying the results available to ensure full reproducibility?

Partly

Is the study design appropriate and is the work technically sound?

Partly

Are the conclusions drawn adequately supported by the results?

Partly

Are sufficient details of methods and analysis provided to allow replication by others?

Partly

Reviewer Expertise:

Virology, immunology, vaccine research, molecular biology, and experimental assay evaluation. My assessment is primarily focused on study design, methodological clarity, data interpretation, and whether the conclusions are adequately supported by the results.

I confirm that I have read this submission and believe that I have an appropriate level of expertise to confirm that it is of an acceptable scientific standard, however I have significant reservations, as outlined above.

# Response

**Published:** 2026-03-31

**Authors:** Francisco Martinez-Perez

**Affiliations:** School of Biology, Industrial University of Santander, Bucaramanga, Santander, Colombia

Dear Reviewer 2,

 We sincerely thank you for your careful and thoughtful evaluation of our manuscript. We appreciate your recognition that the study has methodological value and that the integration of *in silico* design with experimental testing in patient-derived samples represents a strength of the work. We have carefully revised the manuscript in response to your comments and observations.

 Below we summarize the principal revisions made to the manuscript in relation to your concerns.

**1. Clarity of presentation and wording**

**Comment:** You indicated that the manuscript addresses a relevant topic, but that some statements were stronger than the data support and that several sections would benefit from clearer wording and greater precision.

**Response: **We revised the Abstract, Introduction, Results, Discussion, and Conclusions to improve clarity, precision, and overall consistency throughout the manuscript. We also moderated statements that could be interpreted as overstating the findings. In particular, the study is now explicitly framed as an exploratory and methodological contribution, and the wording of the conclusions has been revised to avoid implying clinical validation or broad diagnostic superiority.

**2. Study design and technical soundness**

**Comment: **You considered the overall concept appropriate, but noted that the validation was limited and did not fully support some of the stronger claims made in the manuscript.

**Response: **We agree with this observation and revised the manuscript accordingly. The study is now more clearly described as exploratory in nature. In addition, we added a dedicated Limitations section to explicitly acknowledge the restricted sample size, the context of sample acquisition during the COVID-19 pandemic, and the absence of independent external validation. These changes were introduced to better align the interpretation of the results with the actual scope of the study.

**3. Methodological clarity and reproducibility**

**Comment:** You requested clarification regarding sample-level classification and the comparison of different assay formats, and also noted that the information required for reproducibility was not sufficiently clear and accessible.

**Response: **We substantially revised the Methods section to clarify these points. In particular, we added and reformulated the subsection now entitled “Exploratory Ct-based performance analysis of SARS-CoV-2 RT-qPCR assays.” In this section, we explain that the performance estimates were derived from predefined Ct-based operational criteria applied uniformly across assays, and that these criteria were used solely for internal exploratory comparison. We also clarified how Ct values were pooled and evaluated at the sample level for both single and multiplex assays.

 We additionally revised the Data Availability section to more clearly describe the genomic datasets, sequence alignments, and scripts used in the analyses, and to improve transparency regarding the public repositories associated with these resources. These revisions were intended to facilitate reproducibility within the scope of the study.

**4. Statistical analysis and interpretation**

**Comment: **You noted that the interpretation of sensitivity, specificity, and related metrics should be more cautious, and that their basis and limitations should be explained more explicitly.

**Response: **We carefully revised both the Methods and Results sections in response to this concern. The Ct-based performance analysis is now explicitly presented as exploratory and internal, rather than as a measure of absolute diagnostic accuracy. In the Results section, these values are now described as apparent comparative metrics under a predefined Ct-based classification framework. We also added explicit language stating that, because no independent external clinical validation was available, these metrics should not be interpreted as evidence of clinical diagnostic performance.

**5. Conclusions**

**Comment: **You considered that the conclusions, particularly those related to improved diagnostic performance in multiplex RT-qPCR, were broader than what the dataset could firmly support.

**Response:** We revised the Discussion and Conclusions to moderate these statements. The manuscript no longer presents the findings as demonstrating clinical diagnostic superiority. Instead, the conclusions now emphasize that the evaluated denaturing and dNTP-based conditions were associated with changes in RT-qPCR behaviour under the experimental conditions tested, and that additional studies with larger datasets and independent validation would be required to assess broader applicability.

**6. Additional clarification regarding study scope and clinical information**

**Response: **To further clarify the context of the study, we now explicitly state in the manuscript that this was not a clinical validation study and that access to participant clinical characterization, including symptom severity and extent of infection, was restricted by the informed consent framework and applicable legal requirements. This clarification was added to the Limitations section to explain why these data were not available for further analysis.

 We are grateful for your constructive comments, which helped us improve the manuscript substantially. We believe that the revised version is clearer, more methodologically transparent, more cautious in its interpretation, and better aligned with the exploratory scope of the work.

 Sincerely,

 Francisco Martinez-Perez

 On behalf of all authors

 Industrial University of Santander

# Reviewer report: Reviewer response for version 1

**DOI:** 10.5256/f1000research.121206.r135398

**Published:** 2022-05-16

**Authors:** Andrew D. Beggs

**Affiliations:** 1 Institute of Cancer and Genomic Sciences, University of Birmingham, Birmingham, UK

The authors have set out to design a new primer set to target the ORF8 gene region of the SARS-CoV-2 genome, as well as understand the effect of denaturing agents on the efficiency of detection by reducing secondary structures and increasing RT-QPCR. It is certainly an interesting approach. However I have a few comments and suggestions to further improve the manuscript

1. The abstract is confusing. They state that they design ORF8 primers using E and N as targets but what they actually mean is that they use these as references. Also they state that the denaturing agents reduce secondary structure and increase efficiency but don't really get the results of this across in the abstract
2. They talk in detail about the characterisation in the GISAID/GenBank databases, but don't explain well that the reason they are doing this is to help design the ORF8 gene probes. This should be clarified. 
3. The primer/probe concentrations are not given but should be to increase the reproducibility of the experiments
4. A 2xn table of the primer sets with a set threshold in positive/negative and quoted sensitivity, specificity etc would greatly aid interpretation of the data 
5. The authors state in the discussion that "our study is the first to demonstrate the use of three genes both independently or in multiplex reactions to detect the SARS-CoV-2 virus" - the Thermo Taqpath assay uses ORF, N, and S genes for detection and so is multiplex.
6. In the discussion of their analysis of global SARS-CoV-2 mutations they discuss "thousands of millions" of mutations. This makes it seem like there have been this number of mutations rather than reports of mutations, as there will be genomes submitted that have overlapping mutations between genomes. Some clarification on language would be useful. 
7. In addition a discussion of why they chose to target the ORF8 gene would be useful. I assume it is because of the relatively low mutational rate here, but it would be nice to see this explained. 

Is the work clearly and accurately presented and does it cite the current literature?

Partly

If applicable, is the statistical analysis and its interpretation appropriate?

Partly

Are all the source data underlying the results available to ensure full reproducibility?

Yes

Is the study design appropriate and is the work technically sound?

Yes

Are the conclusions drawn adequately supported by the results?

Partly

Are sufficient details of methods and analysis provided to allow replication by others?

Yes

Reviewer Expertise:

SARS-CoV-2 genetics.

I confirm that I have read this submission and believe that I have an appropriate level of expertise to confirm that it is of an acceptable scientific standard, however I have significant reservations, as outlined above.

# Response

**Published:** 2024-03-14

**Authors:** Francisco Martinez-Perez

**Affiliations:** School of Biology, Industrial University of Santander, Bucaramanga, Santander, Colombia

**Reviewer 1.**

**Andrew D. Beggs**

**Institute of Cancer and Genomic Sciences,**

**University of Birmingham, Birmingham, UK.**

 The authors have set out to design a new primer set to target the *ORF8* gene region of the SARS-CoV-2 genome, as well as understand the effect of denaturing agents on the efficiency of detection by reducing secondary structures and increasing RT-qPCR. It is certainly an interesting approach. However, I have a few comments and suggestions to further improve the manuscript.

**Response: We greatly appreciate the comments, suggestions and corrections made by reviewer 1, which greatly enriched the work.**

 1. The abstract is confusing. They state that they design *ORF8* primers using *E *and *N* as targets but what they actually mean is that they use these as references. Also, they state that the denaturing agents reduce secondary structure and increase efficiency but don't really get the results of this across in the abstract.

**Response: The correction is accepted. The abstract of the manuscript is modified and expanded, providing clarity in the methods and results sections.**

**Abstract**

**Background: **The COVID-19 pandemic, caused by the SARS-CoV-2, can be effectively managed with diagnostic tools such as RT-qPCR. However, it can produce false-negative results due to viral mutations and RNA secondary structures from the target gene sequence.

**Methods: **With High Performance Computing, the complete SARS-CoV-2 genome was obtained from the GenBank/GISAID to generate consensus sequences to design primers/probes for RT-qPCR. *ORF8* gene was selected, meanwhile, *E* and *N* and *RNAse P* were according to CDC protocol. Nasopharyngeal swab samples were collected from patients diagnosed with SARS-CoV-2. Total RNA was purified according MagMAX kit, it was used in single, and multiplex RT-qPCR. To avoid templated secondary structures, compensate nucleotide proportions and improve Ct values, a solution composed of tetraethylammonium chloride and dimethyl sulfoxide and other with corresponding to dNTPs proportions in accordance SARS-CoV-2 genome were included. Sensitivity and specificity according to Ct values were determined with the Caret package in R software.

**Results: **126,576 SARS-CoV-2 genomes from January to December 2020 comprised a database. From this, a region near of 5' *ORF8* gene showed three stem-loops was used for primers/FAM-probe. 49 samples were obtained, from them, 22 were positive to gene selected regions. Interestingly, samples from October to November 2020 were positive for all regions, however, in January 2021 different results were observed in *ORF8*. An improvement in Ct with the adjuvant solutions was determined in all samples with others SARS-CoV-2 primers/probes, for both single and multiplex RT-qPCR. The inclusion of the denaturing solution in single RT-qPCR increased its sensitivity with respect to the commercial method, while in multiplex the opposite was generated.

**Conclusions: **Including adjuvant solutions to prevent the formation of RNA secondary structures and the adjustment of the nucleotide ratios of SARS-CoV-2 improved single and multiplex RT-qPCR for viral identification, demonstrating its potential application in health public.

 2. They talk in detail about the characterization in the GISAID/GenBank databases, but don't explain well that the reason they are doing this is to help design the ORF8 gene probes. This should be clarified.

**Response: Clarity is provided in the methods to use of databases for *ORF8* design.**

 Due to the appearance of mutations in several genes of the SARS-CoV-2 reference genome that could generate false negatives with the primers and probes authorized for identification by RT-qPCR. A region coding for the accessory protein ORF8 was selected, hypothesizing that it did not have a high mutation frequency. Therefore, monthly consensus sequences were generated to determine its identity pattern with respect to new genomes.

 3. The primer/probe concentrations are not given but should be to increase the reproducibility of the experiments.

**Response: The concentration in nmole of the primers/probes is reported below. It could also be included in Table 1.**

 4. A 2xn table of the primer sets with a set threshold in positive/negative and quoted sensitivity, specificity etc. would greatly aid interpretation of the data.

**Response: The calculation of the sensitivity, specificity, Positive Predictive Value (PPV) and Negative Predictive Value (NPV) of the viral identification tests of the participants was included in the manuscript.**

**Methodology**

**Calculation of sensitivity, specificity and predictive values of SARS-CoV-2** **.**

 To calculate the sensitivity, specificity, Positive Predictive Value (PPV) and Negative Predictive Value (NPV) of each of the reactions with the single and multiplex primers used, a script was implemented in R using the Caret package to calculate the values generated by each RT-qPCR with their respective primers/probes (https://github.com/GenomicUIS/Sensitivity-specificity-PPV-and-NPV-for-SARS-CoV-2.git) (Kuhn, 2008; R Core Team 2020). Cycle threshold (Ct) was defined for each of the primers in the single reactions of each of the processed samples, where, the value of 18-35 Ct was considered true positive, Ct 35 > false positive, Ct 18 < true negative. Subsequently, the Cts values obtained were pooled and evaluated per sample. Therefore, if the result generated by a set of primers/probes was at the above-mentioned threshold, it was considered a true positive identification. Whereas, results outside the threshold were considered false negative and indeterminate. Cts were identical for multiplex RT-qPCR reactions, but the results were evaluated as a whole.

 R Core Team. R: A language and environment for statistical computing. *R Foundation for Statistical Computing*. 2020; Vienna, Austria. URL https://www.R-project.org/.

 Kuhn M: Building Predictive Models in R Using the caret Package. *J Stat Softw*. 2008; 28(5): 1–26. Doi: 10.18637/jss.v028.i05

**Results**

**Sensitivity, specificity and predictive values of SARS-CoV-2**

 The performance of single and multiplex RT-qPCRs with the solutions used showed that single denaturing was more sensitive than the commercial solution, but the latter is 33% more specific than the former. As for the multiplex reactions, the commercial solution showed non-significant difference in sensitivity with respect to the denaturing solution. Similarly, the specificity of the single reactions was better for the commercial solution with respect to the denaturing solution, but in the multiplex reactions this difference was not evident in the detection of true negatives as both solutions yielded results of 0. dNTPs were not evaluated due to the number of positive results (Table 4).

**Discussion**

 The reagents and methods described herein allow for an immediate, facile, and cost-effective detection of the SARS-CoV-2 virus; which was demonstrated by comparing the Cts of the positive results used to determine sensitivity and specificity with those published for other countries (Arakawa *et al.,* 2024; Aranha *et al*., 2021; Chen *et al*., 2022).

 Arakawa Y, Nishida Y, Sakanashi D, *et al*.: Clinical evaluation of a modified SARS-CoV-2 rapid molecular assay, ID NOW ^TM^ COVID-19 2.0. *J. Infect. Chemother*. 2024; S1341-321X(24): 00073-4. Doi: 10.1016/j.jiac.2024.02.032.

 Aranha C, Patel V, Bhor V, *et al*.: Cycle threshold values in RT-PCR to determine dynamics of SARS-CoV-2 viral load: An approach to reduce the isolation period for COVID-19 patients. *J. Med. Virol*. 2021; 93(12): 6794-6797. Doi: 10.1002/jmv.27206.

 Chen YY, Shen X, Wang YJ, *et al*.: Evaluation of the cycle threshold values of RT-PCR for SARS-CoV-2 in COVID-19 patients in predicting epidemic dynamics and monitoring surface contamination. *J. Infect. Public. Health*. 2022; 15(12): 1494-1496. Doi: 10.10 16/j.jiph.2022.11.012.

 Based on our findings, we concluded that multiplex RT-qPCR is an optimal solution for the aforementioned limitations, even though the Cts used to establish sensitivity and specificity did not show a difference in some samples between commercial methods compared to the one proposed here.

 5. The authors state in the discussion that "our study is the first to demonstrate the use of three genes both independently or in multiplex reactions to detect the SARS-CoV-2 virus" -the Thermo Taqpath assay uses ORF, N, and S genes for detection and so is multiplex.

**Response: To our knowledge, at the time the experiments for this study were conducted (19 September 2020 to 30 January 2021), there was no kit that performed viral identification with three genes.**

 According to the official website of Thermo Fisher Scientific, the Taqpath kit is based on sequences obtained from GenBank and GISAID as of 7 May 2021 (https://www.thermofisher.com/co/en/home/clinical/clinical-genomics/pathogen-detection-solutions/covid-19-sars-cov-2/multiplex.html), and subsequently obtained a letter of approval for use by the Food and Drug Administration (FDA) on 18 May 2022 (https://www.fda.gov/media/136113/download). Our public database was generated by HPC between January and December 2020 (https://zenodo.org/records/6337537).

 6. In the discussion of their analysis of global SARS-CoV-2 mutations they discuss "thousands of millions" of mutations. This makes it seem like there have been this number of mutations rather than reports of mutations, as there will be genomes submitted that have overlapping mutations between genomes. Some clarification on language would be useful.

**Response: The correction is accepted, and clarity is given in the manuscript.**

 Our analysis of the global SARS-CoV-2 mutation patterns based on the GenBank and GISAID databases indicated that most mutations began in March 2020, and subsequently increased significantly, relative to the SARS-CoV-2 reference genome (GenBank access: NC_045512.2).

 7. In addition, a discussion of why they chose to target the *ORF8* gene would be useful. I assume it is because of the relatively low mutational rate here, but it would be nice to see this explained.

**Response: Correction accepted.**

 Using the SARS-CoV-2 (NC_045512) reference genome and consensus sequences from January to April 2020 (Zhu et al., 2020), an approximately 150-base pair (bp) region of the ORF8 gene was selected based on the secondary structure of its RNA transcript, which in turn was predicted using the algorithms proposed by Zuker (Zuker & Jacobson, 1998) via the Mflod software (RRID:SCR_001360) (Zuker, 2003). Thus, the last set containing the codons of the central region of the *ORF8* gene, which encodes the secretion protein ORF8, was chosen because it allows proper viral adhesion to the host cell (Chan et al., 2020).

 Chan JF, Kok KH, Zhu Z, *et al*.: Genomic characterization of the 2019 novel human-pathogenic coronavirus isolated from a patient with atypical pneumonia after visiting Wuhan. *Emerg. Microbes. Infect*. 2020; 9(1): 221-236. DOI: 10.1080/22221751.2020.1719902.
